# Supplementary material for: A flexible approach for causal inference with multiple treatments and clustered survival outcomes
Source: Stat Med. 2022 Aug 10;41(25):4982–99. doi: 10.1002/sim.9548 (PMC9588538; doi:10.1002/sim.9548)
Supplement: Supplementary file 1 — Data S1: Supplementary material [file SIM-41-4982-s001.pdf]

Web-based Supplementary Materials for "A flexible approach for causal inference with multiple treatments and clustered survival outcomes" by Hu et al.

email: liangyuan.hu@rutgers.edu

S1 Posterior distributions of μ_{lh}, σ^2, b_k, α_k and τ^2 in riAFT-BART

1) For the posterior of σ^2, since we have $\sigma^2 \sim IG\left(\frac{\nu}{2}, \frac{\nu\lambda}{2}\right)$, we obtain

$$
\begin{aligned}
&P\left(\sigma^2 \,|\, y_{ik}^{cent,c}, \boldsymbol{X}_{ik}, A_{ik}, V_k, b_k, \tau^2, \alpha_k, \{\mathcal{W}_H, \mathcal{M}_H\}\right) \\
&\propto P\left(y_{ik}^{cent,c} \,|\, \boldsymbol{X}_{ik}, A_{ik}, V_k, b_k, \{\mathcal{W}_H, \mathcal{M}_H\}\right) P\left(\sigma^2\right) \\
&\propto \left\{\prod_{k=1}^{K}\prod_{i=1}^{n_k} \left(\sigma^2\right)^{-\frac{1}{2}} \exp\left[-\frac{\left(y_{ik}^{cent,c} - \hat{f}(\boldsymbol{X}_{ik}, A_{ik}) - b_k\right)^2}{2\sigma^2}\right]\right\} \left(\sigma^2\right)^{-\left(\frac{\nu}{2}+1\right)} \exp\left[-\frac{\nu\lambda}{2\sigma^2}\right] \\
&\propto \left(\sigma^2\right)^{-\left(\frac{N+\nu}{2}+1\right)} \exp\left[-\frac{\sum_{k=1}^{K}\sum_{i=1}^{n_k}\left(y_{ik}^{cent,c} - \hat{f}(\boldsymbol{X}_{ik}, A_{ik}) - b_k\right)^2 + \nu\lambda}{2\sigma^2}\right]
\end{aligned}
$$

$$
\begin{aligned}
&P\left(\sigma^2 \,|\, y_{ik}^{cent,c}, \boldsymbol{X}_{ik}, A_{ik}, V_k, \tau^2, \alpha_k, b_k, \{\mathcal{W}_H, \mathcal{M}_H\}\right) \\
&\sim IG\left(\frac{N+\nu}{2}, \frac{\sum_{k=1}^{K}\sum_{i=1}^{n_k}\left(y_{ik}^{cent,c} - \hat{f}(\boldsymbol{X}_{ik}, A_{ik}) - b_k\right)^2 + \nu\lambda}{2}\right)
\end{aligned}
$$

2) For the posterior of the random intercept b_k

$$
\begin{aligned}
&P\left(b_k \,|\, y_{ik}^{cent,c}, \boldsymbol{X}_{ik}, A_{ik}, V_k, \tau^2, \alpha_k, \sigma^2, \{\mathcal{W}_H, \mathcal{M}_H\}\right) \\
&\propto P\left(y_{ik}^{cent,c} \,|\, \boldsymbol{X}_{ik}, A_{ik}, V_k, \{\mathcal{W}_H, \mathcal{M}_H\}, \sigma^2, b_k\right) P\left(b_k \,|\, \tau^2, \alpha_k\right) \\
&\propto \left[\prod_{i=1}^{n_k} \exp\left\{-\frac{\left(y_{ik}^{cent,c} - \hat{f}(\boldsymbol{X}_{ik}, A_{ik}) - b_k\right)^2}{2\sigma^2}\right\}\right] \exp\left\{-\frac{b_k^2}{\alpha_k \tau^2}\right\} \\
&\propto \exp\left\{-\frac{\sum_{i=1}^{n_k}\left(y_{ik}^{cent,c} - \hat{f}(\boldsymbol{X}_{ik}, A_{ik}) - b_k\right)^2}{2\sigma^2}\right\} \exp\left\{-\frac{b_k^2}{2\alpha_k \tau^2}\right\} \\
&\propto \exp\left\{-\frac{\left(n_k\tau^2\alpha_k + \sigma^2\right) b_k^2 - 2\tau^2 b_k \alpha_k \sum_{i=1}^{n_k}\left(y_{ik}^{cent,c} - \hat{f}(\boldsymbol{X}_{ik}, A_{ik})\right)}{2\sigma^2\tau^2\alpha_k}\right\} \\
&\propto \exp\left\{-\frac{\left(b_k - \frac{\tau^2\alpha_k \sum_{i=1}^{n_k}\left(y_{ik}^{cent,c} - \hat{f}(\boldsymbol{X}_{ik}, A_{ik})\right)}{n_k\tau^2\alpha_k + \sigma^2}\right)^2}{2\frac{\sigma^2\tau^2\alpha_k}{n_k\tau^2\alpha_k + \sigma^2}}\right\}.
\end{aligned}
$$

$$
P\left(b_k \,|\, y_{ik}^{cent,c}, \boldsymbol{X}_{ik}, A_{ik}, V_k, \tau^2, \alpha_k, \sigma^2, \{\mathcal{W}_H, \mathcal{M}_H\}\right) \sim N\left(\frac{\tau^2\alpha_k \sum_{i=1}^{n_k}\left(y_{ik}^{cent,c} - \hat{f}(\boldsymbol{X}_{ik}, A_{ik})\right)}{n_k\tau^2\alpha_k + \sigma^2}, \frac{\sigma^2\tau^2\alpha_k}{n_k\tau^2\alpha_k + \sigma^2}\right)
$$

3) For the posterior of α_k, used for parameter expansion

$$
\begin{aligned}
&P\left(\alpha_k \,|\, y_{ik}^{cent,c}, \boldsymbol{X}_{ik}, A_{ik}, V_k, \tau^2, b_k, \sigma^2, \{\mathcal{W}_H, \mathcal{M}_H\}\right) \\
&\propto \left\{\prod_{k=1}^{K} P\left(b_k \,|\, \tau^2, \alpha_k\right)\right\} P(\alpha_k) \\
&\propto \exp\left\{-\frac{\sum_{k=1}^{K} b_k^2}{2\alpha_k\tau^2}\right\} \left\{\alpha_k^{-2} \exp\left(-\frac{1}{\alpha_k}\right)\right\} \\
&\propto \alpha_k^{-(1+1)} \exp\left\{-\frac{1}{\alpha_k}\left(1 + \frac{\sum_{k=1}^{K} b_k^2}{2\tau^2}\right)\right\}.
\end{aligned}
$$

$$
P\left(\alpha_k \,|\, y_{ik}^{cent,c}, \boldsymbol{X}_{ik}, A_{ik}, V_k, \tau^2, b_k, \sigma^2, \{\mathcal{W}_H, \mathcal{M}_H\}\right) \sim IG\left(1, 1 + \frac{\sum_{k=1}^{K} b_k^2}{2\tau^2}\right)
$$

4) For the posterior of τ^2

$$\begin{aligned}
&P\left(\tau^2 \,|\, y_{ik}^{cent,c}, \boldsymbol{X}_{ik}, A_{ik}, V_k, b_k, \alpha_k, \sigma^2, \{\mathcal{W}_H, \mathcal{M}_H\}\right) \\
&\propto P\left(y_{ik}^{cent,c} \,|\, \boldsymbol{X}_{ik}, A_{ik}, V_k, \{\mathcal{W}_H, \mathcal{M}_H\}, \sigma^2, b_k, \alpha_k\right) P(\tau^2) \\
&\propto \left\{\prod_{k=1}^{K} P\left(b_k \,|\, \tau^2, \alpha_k\right)\right\} P(\tau^2) \\
&\propto (\tau^2)^{-K/2} \exp\left\{-\frac{\sum_{k=1}^{K} b_k^2}{2\alpha_k \tau^2}\right\} (\tau^2)^{-2} \exp\left(-\frac{1}{\tau^2}\right) \\
&\propto (\tau^2)^{-K/2+1+1} \exp\left(-\frac{\sum_{k=1}^{K} b_k^2 + 2\alpha_k}{2\alpha_k \tau^2}\right).
\end{aligned}$$

$$P\left(\tau^2 \,|\, y_{ik}^{cent,c}, \boldsymbol{X}_{ik}, A_{ik}, V_k, b_k, \alpha_k, \sigma^2, \{\mathcal{W}_H, \mathcal{M}_H\}\right) \sim IG\left(\frac{K}{2}+1, \frac{\sum_{k=1}^{K} b_k^2 + 2\alpha_k}{2\alpha_k}\right)$$

5) For the posterior of μ_{lh}, we first draw the Gibbs sample of $b_k, \alpha_i, \tau^2, \sigma^2$ seperately from their respective posterior distribution. Then using the updated b_k, we obtain $\tilde{Y}_{ik}^{c,c} = Y_{ik}^{c,c} - b_k$. Now $\tilde{Y}_{ik}^{c,c} \mid \boldsymbol{X}_{ik}$ can be viewed as a BART model. We have $\tilde{R}_{lh} \mid g\left(\mathbf{X}_{ik}, T_j, \mathbf{M}_j\right), \sigma \sim N(\mu_{lh}, \sigma^2), \mu_{lh} \mid \{\mathcal{W}_H\} \sim N(\mu_\mu, \sigma_\mu^2)$ where $\tilde{R}_{lh}^{c,c} = Y_{ik}^{c,c} - b_k - \sum_{w \neq h} g\left(\mathbf{X}_{ik}, \mathcal{W}_w, \mathcal{M}_w\right)$

$$\begin{aligned}
&P\left(\mu_{lh} \,|\, y_{ik}^{cent,c}, \boldsymbol{X}_{ik}, A_{ik}, V_k, b_k, \tau^2, \alpha_k, \sigma^2, \{\mathcal{W}_H\}\right) \\
&\propto P(\mu_{lh} \mid \{\mathcal{W}_H\}, \sigma^2, \tilde{R}_{lh}) \\
&\propto P(\tilde{R}_{lh} \mid \{\mathcal{W}_H\}, \mu_{lh}, \sigma) P(\mu_{lh} \mid \{\mathcal{W}_H\}) \\
&\propto \exp\left\{-\frac{\sum_i (\tilde{r}_{lh} - \mu_{lh})^2}{2\sigma^2}\right\} \exp\left\{-\frac{(\mu_{lh} - \mu_\mu)^2}{2\sigma_\mu^2}\right\} \\
&\propto \exp\left\{-\frac{\sigma_\mu^2 \sum_i (\tilde{r}_{lh}^2 + \mu_{lh} - 2\mu_{lh}\tilde{r}_{lh}) + \sigma^2(\mu_{lh}^2 + \mu_\mu^2 - 2\mu_\mu \mu_{lh})}{2\sigma^2\sigma_\mu^2}\right\} \\
&\propto \exp\left\{-\frac{\sigma_\mu^2 n_i \mu_{lh} - 2\sigma_\mu^2 \mu_{lh} \sum_i \tilde{r}_{lh} + \sigma^2 \mu_{lh}^2 - 2\sigma^2 \mu_\mu \mu_{lh})}{2\sigma^2\sigma_\mu^2}\right\} \\
&\propto \exp\left\{-\frac{(n_i\sigma_\mu^2 + \sigma^2)\mu_{lh}^2 - 2(\sigma_\mu^2 \sum_i \tilde{r}_{lh} + \sigma^2\mu_\mu)\mu_{lh}}{2\sigma^2\sigma_\mu^2}\right\} \\
&\propto \exp\left\{-\frac{(\mu_{lh} - \frac{\sigma_\mu^2 \sum_i \tilde{r}_{lh} + \sigma^2\mu_\mu}{n_i\sigma_\mu^2 + \sigma^2})^2}{\frac{2\sigma^2\sigma_\mu^2}{n_i\sigma_\mu^2 + \sigma^2}}\right\}
\end{aligned}$$

$$P\left(\mu_{lh} \,|\, y_{ik}^{cent,c}, \boldsymbol{X}_{ik}, A_{ik}, V_k, b_k, \tau^2, \alpha_k, \sigma^2, \{\mathcal{W}_H\}\right) \sim N\left(\frac{\sigma_\mu^2 \sum_i \tilde{r}_{lh} + \sigma^2\mu_\mu}{n_i\sigma_\mu^2 + \sigma^2}, \frac{\sigma^2\sigma_\mu^2}{n_i\sigma_\mu^2 + \sigma^2}\right)$$

S2 Technical details for sensitivity analysis in Section 3.2

The derivation of the bias formula $\text{Bias}(a_j, a_{j'} \mid \boldsymbol{x}, v)$ in equation (5) follows the proof of Theorem 2.1 in Hu et al. (2022).

Under the weak unconfoundedness assumption (A2), ignoring individual-level unmeasured confounding will lead to the following bias in the estimate of the causal effect $CATE_{a_j,a_{j'}}$,

$$\begin{aligned}\text{Bias}(a_j, a_{j'} \mid \boldsymbol{x}, v) &= E[T|A=a_j, \boldsymbol{X}=\boldsymbol{x}, V=v] - E[T|A=a_{j'}, \boldsymbol{X}=\boldsymbol{x}, V=v] \\ &\quad -E\left[T(a_j) - T(a_{j'}) \mid \boldsymbol{X}=\boldsymbol{x}, V=v\right].\end{aligned}$$

To simplify notation, we will use $E[\cdot \mid a, \boldsymbol{x}, v]$ to denote $E[\cdot \mid A=a, \boldsymbol{X}=\boldsymbol{x}, V=v]$. Applying the law of total expectation to $E[T(a_j \mid \boldsymbol{X}=\boldsymbol{x}, V=v)]$, we have

$$E[T(a_j) \mid \boldsymbol{X}=\boldsymbol{x}, V=v] = \sum_{l=0}^{J} p_l E[T(a_j) \mid a_l, \boldsymbol{X}=\boldsymbol{x}, V=v].$$

Similary,

$$E\left[T(a_{j'}) \mid \boldsymbol{X}=\boldsymbol{x}, V=v\right] = \sum_{l=0}^{J} p_l E[T(a_j) \mid \boldsymbol{X}=\boldsymbol{x}, V=v].$$

Hence,

$$\begin{aligned}E\left[T(a_j) - Y(a_{j'}) \mid \boldsymbol{x}, v\right] &= p_0 E\left[T(a_j) - T(a_{j'}) \mid a_0, \boldsymbol{x}, v\right] + \ldots + p_J E\left[T(a_j) - T(a_{j'}) \mid a_J, \boldsymbol{x}, v\right] \\ &\quad + p_j E\left[T(a_j) - T(a_{j'}) \mid a_j, \boldsymbol{x}, v\right] + p_{j'} E\left[T(a_j) - T(a_{j'}) \mid a_{j'}, \boldsymbol{x}, v\right]. \quad (1)\end{aligned}$$

Repeatedly using $E[T(a_l) \mid a_l, \boldsymbol{x}, v] = E[T \mid a_l, \boldsymbol{x}, v], \forall l \in \{1, \ldots, J\}$ to rewrite the last two items of the RHS of the equation (1), we have

$$\begin{aligned}& p_j E\left[T(a_j) - T(a_{j'}) \mid a_j, \boldsymbol{x}, v\right] + p_{j'} E\left[T(a_j) - T(a_{j'}) \mid a_{j'}, \boldsymbol{x}, v\right] \\ = \; & p_j E[T(a_j) \mid a_j, \boldsymbol{x}, v] - p_j E\left[T(a_{j'}) \mid a_j, \boldsymbol{x}, v\right] + p_{j'} E\left[T(a_j) \mid a_{j'}, \boldsymbol{x}, v\right] - p_{j'} E\left[T(a_{j'}) \mid a_{j'}, \boldsymbol{x}, v\right] \\ = \; & p_j E[T \mid a_j, \boldsymbol{x}, v] - p_{j'} E\left[T \mid a_{j'}, \boldsymbol{x}, v\right] + p_{j'} E\left[T(a_j) \mid a_{j'}, \boldsymbol{x}, v\right] - p_j E\left[T(a_{j'}) \mid a_j, \boldsymbol{x}, v\right] \\ = \; & p_j \left\{E[T \mid a_j, \boldsymbol{x}, v] - E\left[T \mid a_{j'}, \boldsymbol{x}, v\right]\right\} + p_j E\left[T \mid a_{j'}, \boldsymbol{x}, v\right] - p_{j'} E\left[T \mid a_{j'}, \boldsymbol{x}, v\right] \\ & -p_j E\left[T(a_{j'}) \mid a_j, \boldsymbol{x}, v\right] + p_{j'} E\left[T(a_j) \mid a_{j'}, \boldsymbol{x}, v\right] \\ = \; & p_j \left\{E[T \mid a_j, \boldsymbol{x}, v] - E\left[T \mid a_{j'}, \boldsymbol{x}, v\right]\right\} + p_j \left\{E\left[T(a_{j'}) \mid a_{j'}, \boldsymbol{x}, v\right] - E\left[T(a_{j'}) \mid a_j, \boldsymbol{x}, v\right]\right\} \\ & +p_{j'} \left\{E\left[T(a_j) - T(a_{j'}) \mid a_{j'}, \boldsymbol{x}, v\right]\right\} \\ = \; & p_j \left\{E[T \mid a_j, \boldsymbol{x}, v] - E\left[T \mid a_{j'}, \boldsymbol{x}, v\right]\right\} + p_j c(a_{j'}, a_j, \boldsymbol{x}, v) + p_{j'} \left\{E\left[T(a_j) - T(a_{j'}) \mid a_{j'}, \boldsymbol{x}, v\right]\right\} \quad (2)\end{aligned}$$

Let $\tilde{p} = 1 - p_j - p_{j'}$. By rewriting $p_{j'} \left\{E\left[T(a_j) - T(a_{j'}) \mid a_{j'}, \boldsymbol{x}, v\right]\right\}$ in equation (2), we have

$$\begin{aligned}& p_{j'} \left\{E\left[T(a_j) - T(a_{j'}) \mid a_{j'}, \boldsymbol{x}, v\right]\right\} \\ = \; & (1 - p_j - \tilde{p}) \left\{E[T \mid a_j, \boldsymbol{x}, v] - E\left[T \mid a_{j'}, \boldsymbol{x}, v\right] + E\left[T(a_j) \mid a_{j'}, \boldsymbol{x}, v\right] - E[T(a_j) \mid a_j, \boldsymbol{x}, v]\right\} \\ = \; & (1 - p_j) \left\{E[T \mid a_j, \boldsymbol{x}, v] - E\left[T \mid a_{j'}, \boldsymbol{x}, v\right]\right\} - (1 - p_j - \tilde{p}) c(a_j, a_{j'}, \boldsymbol{x}, v) \\ & -\tilde{p} \left\{E[T \mid a_j, \boldsymbol{x}, v] - E\left[T \mid a_{j'}, \boldsymbol{x}, v\right]\right\} \\ = \; & (1 - p_j) \left\{E[T \mid a_j, \boldsymbol{x}, v] - E\left[T \mid a_{j'}, \boldsymbol{x}, v\right]\right\} - p_{j'} c(a_j, a_{j'}, \boldsymbol{x}, v) \\ & -(1 - p_j - p_{j'}) \left\{E[T(a_j) \mid a_j, \boldsymbol{x}, v] - E\left[T(a_{j'}) \mid a_{j'}, \boldsymbol{x}, v\right]\right\}.\end{aligned}$$

Taken together,

$$
\begin{aligned}
\text{Bias}(a_j, a_{j'} \mid \boldsymbol{x}, v) &= \sum_{m: a_m \in \mathscr{A} \setminus \{a_j, a_{j'}\}} -p_m E\left[T(a_j) - T(a_{j'}) \mid a_m, \boldsymbol{x}, v\right] - p_j c(a_{j'}, a_j, \boldsymbol{x}, v) + p_{j'} c(a_j, a_{j'}, \boldsymbol{x}, v) \\
&\quad + \sum_{m: a_m \in \mathscr{A} \setminus \{a_j, a_{j'}\}} p_m \left\{E\left[T(a_j) \mid a_j, \boldsymbol{x}, v\right] - E\left[T(a_{j'}) \mid a_{j'}, \boldsymbol{x}, v\right]\right\} \\
&= -p_j c(a_{j'}, a_j, \boldsymbol{x}, v) + p_{j'} c(a_j, a_{j'}, \boldsymbol{x}, v) \\
&\quad - \sum_{m: a_m \in \mathscr{A} \setminus \{a_j, a_{j'}\}} p_m \left\{c(a_{j'}, a_m, \boldsymbol{x}, v) - c(a_j, a_m, \boldsymbol{x}, v)\right\}.
\end{aligned}
$$

We now show how we arrive at equation (6) for adjusting the responses so that the bias in equation (5) due to individual-level unmeasured confounding will be effectively removed from the confounding function adjusted treatment effect estimate.

Because the causal effect is defined as the between-group difference in mean potential outcomes and is estimated based on the observed outcomes. To correct the bias in equation (5) due to individual-level unmeasured confounding, we adjust the actual survival time T of an individual who received treatment a_j as

$$
\log T^{CF} = \log T - \left[E\left\{\log T(a_j) \mid a_j, \boldsymbol{x}, v\right\} - E\left\{\log T(a_j) \mid \boldsymbol{x}, v\right\}\right]. \tag{3}
$$

Let $p_j \equiv P(A = a_j \mid \boldsymbol{X} = \boldsymbol{x}, V = v)$ and $E\left[\cdot \mid a_j, \boldsymbol{x}, v\right] \equiv E\left[\cdot \mid A = a_j, \boldsymbol{X} = \boldsymbol{x}, V = v\right], \forall a_j \in \mathscr{A}$. By applying the law of total expectation to $E\left[T(a_j) \mid \boldsymbol{x}, v\right]$, we can rewrite the second quantity of the RHS of equation (3) as

$$
\begin{aligned}
& E\left[\log T(a_j) \mid a_j, \boldsymbol{x}, v\right] - E\left[\log T(a_j) \mid \boldsymbol{x}, v\right] \\
=\ & E\left[\log T(a_j) \mid a_j, \boldsymbol{x}, v\right] - \sum_{m=1}^{J} p_m E\left[\log T(a_j) \mid a_m, \boldsymbol{x}, v\right] \\
=\ & (1 - p_j) E\left[\log T(a_j) \mid a_j, \boldsymbol{x}, v\right] - \sum_{m \neq j}^{J} p_m E\left[\log T(a_j) \mid a_m, \boldsymbol{x}, v\right] \\
=\ & \sum_{m \neq j}^{J} p_m \left\{E\left[\log T(a_j) \mid a_j, \boldsymbol{x}, v\right] - E\left[\log T(a_j) \mid a_m, \boldsymbol{x}, v\right]\right\} \\
=\ & \sum_{m \neq j}^{J} p_m c(a_j, a_m, \boldsymbol{x}, v).
\end{aligned}
$$

This implies that we will replace T with T^{CF} as $\log T^{CF} = \sum_{m \neq j}^{J} p_m c(a_j, a_m, \boldsymbol{x}, v)$, which is equation (6).

Now we prove that replacing T with T^{CF} removes the bias in equation (5). Consider the causal effect between any pair of treatments a_j and $a_{j'}$. Using the adjusted survival times T^{CF}, the

estimate of the causal effect is

$$
\begin{aligned}
& E\left[\log T^{CF} \mid a_j, \boldsymbol{x}, v\right] - E\left[\log T^{CF} \mid a_{j'}, \boldsymbol{x}, v\right] \\
= \; & E\left[\left(\log T - \sum_{m \neq j}^{J} p_m c(a_j, a_m, \boldsymbol{x}, v)\right) \mid a_j, \boldsymbol{x}, v\right] - E\left[\left(\log T - \sum_{m \neq j'}^{J} p_m c(a_{j'}, a_m, \boldsymbol{x}, v)\right) \mid a_{j'}, \boldsymbol{x}, v\right] \\
= \; & E(\log T \mid a_j, \boldsymbol{x}, v) - E(\log T \mid a_{j'}, \boldsymbol{x}, v) \\
& \underbrace{+ p_j c(a_{j'}, a_j, \boldsymbol{x}, v) - p_{j'} c(a_j, a_{j'}, \boldsymbol{x}, v) + \sum_{m: m \in \mathscr{A} \setminus \{a_j, a_{j'}\}} p_m \left\{c(a_{j'}, a_m, \boldsymbol{x}, v) - c(a_j, a_m, \boldsymbol{x}, v)\right\}}_{\text{–Bias in equation (5)}} .
\end{aligned}
$$

Because the survival time T can be right censored, we can replace the centered complete-data survival time $\log y_{ik}^{cent,c}$ used in our riAFT-BART sampling algorithm for treatment effect estimation with adjusted $\log y_{ik}^{CF}$ as in equation (6). Running riAFT-BART with $\log y_{ik}^{CF}$ will effectively remove the bias in equation (5) from the confounding function adjusted treatment effect estimate.

S3 Additional simulations from different survival models

S3.1 The Pareto survival curves

We first generate the survival data from a Pareto distribution to assess the performance of our proposed method among data not adhering to the AFT model. We generate the counterfactual survival times from a Pareto survival curve,

$$
S_{ik}(t) = \frac{\lambda^{\theta_{a_j}}}{\left\{\left(\beta_{a_j}^{L}, \beta_{a_j}^{NL}; \boldsymbol{X}_{ik}, \boldsymbol{G}_{ik}, b_k\right) t\right\}^{\theta_{a_j}}} \tag{4}
$$

where $\beta_{a_j}^{L}$ is a treatment-specific vector of coefficients for $\boldsymbol{X}_{ik}$ and $\beta_{a_j}^{NL}$ for $\boldsymbol{G}_{ik}$, the nonlinear transformations and higher-order terms of the predictors $\boldsymbol{X}_{ik}$, $\forall a_j \in \{1, 2, 3\}$. The parameter λ was set to 0.005, $b_k \sim N(0, 4^2)$, and $\theta_{a_j} = \{2, 4, 3\}$ for $a_j = 1, 2, 3$. Following the inverse transformation method using the cumulative baseline hazard function (Bender et al., 2005), we generate three sets of non-parallel response surfaces:

$$
T_{ik}\left(a_j\right) = \lambda \exp\left[\frac{-\log U \exp\left(-\boldsymbol{X}_{ik}\beta_{a_j}^{L} - \boldsymbol{G}_{ik}\beta_{a_j}^{NL} - b_k\right)}{\theta_{a_j}}\right], \tag{5}
$$

for $a_j \in \{1, 2, 3\}$, where U is a random variable following the uniform distribution on the interval $[0, 1]$. Observed and uncensored survival times are generated as $T_{ik} = \sum_{a_j \in \{1,2,3\}} T_{ik}(a_j) I(A_{ik} = a_j)$. We further generated censoring time C independently from an exponential distribution with the rate parameter selected to induce two different censoring rates: 10% and 40%. Detailed model specifications for the outcomes, as in model (5), are given in Table 1. The parameter setup is guided by the NCDB data in our case study so that the resulting Kaplan-Meier survival curves (Web Figure 1) are representative of those in the NCDB data.

Web Table 1: Coefficients $\beta^{L}_{a_j}$ and $\beta^{NL}_{a_j}$ of the T-model(5), $a_j \in \{1, 2, 3\}$ to generate counterfactual survival times from the Pareto survival curves.

	T-model		
Variables	$a_j = 1$	$a_j = 2$	$a_j = 3$
x_1	0.2	0.2	0.3
x_2	-0.3	0.2	0.2
x_3	-0.4	-0.2	0.3
x_4	0	0	0
x_5	0	0	0
x_6	0.2	-0.4	0.2
x_7	0.3	-0.3	0.5
x_8	0.4	0.3	0.3
x_9	0.5	0.2	0.2
x_{10}	0.6	0.6	0.1
x_1^2	0.2	0.2	0.1
x_1^3	0.1	0.1	0.
x_2^2	-0.1	0.2	-0.2
$x_2^2 x_5$	0	0	0
$x_2 x_4$	0	0	0
$x_2^2 x_4$	0	0	0
$x_2 x_3$	0.3	-0.2	0.3
$x_2 x_5$	0	0	0
$x_2 x_3^2$	0.4	-0.2	0.3
$x_2 x_4 x_5$	0	0	0
$x_1 x_2 x_3$	-0.1	-0.2	0.2
$\sin(2\pi x_1 x_3)$	0	0	0
$\sin(\pi x_1 x_3)$	0	0	0
$\sin(\pi x_4 x_5)$	0	-0.6	0.5
$\sin(2\pi x_4 x_5)$	0.6	0	0

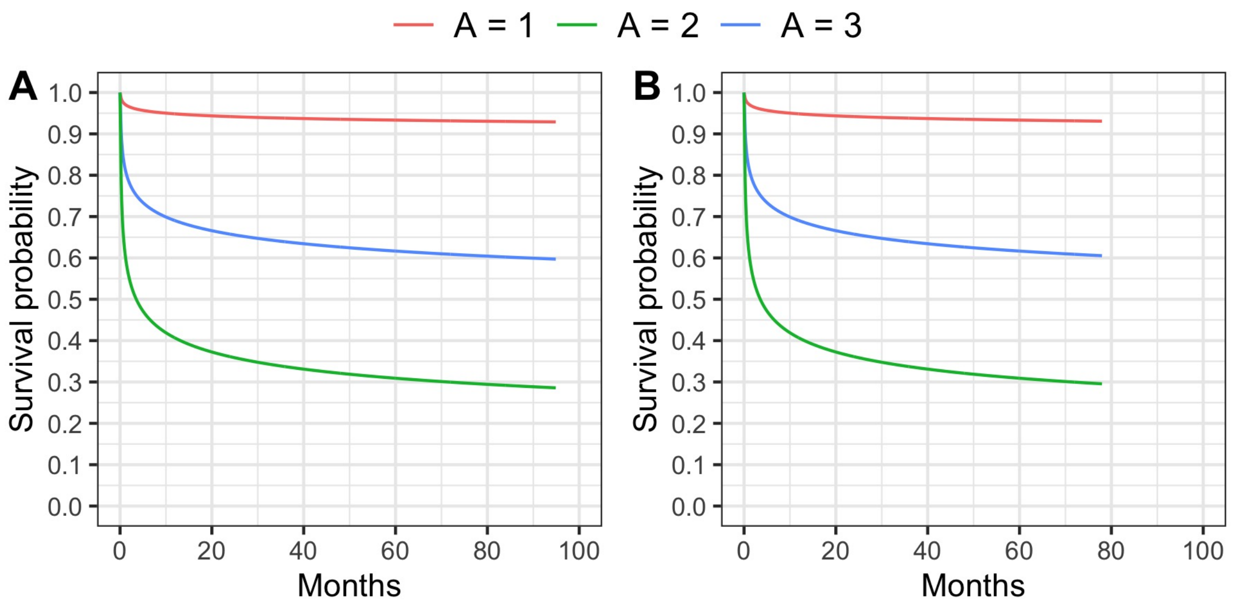

Web Figure 1: Kaplan-Meier survival curves by treatment group. The survival times were generated from the Pareto distribution (equation(5)). Panels A represents the scenario of 10% censoring. Panels B represents the scenario of 40% censoring.

Web Figure 2 and Web Figure 3 respectively display boxplots of relative biases in three treatment effect estimates $\widehat{\text{CATE}}_{1,2}$, $\widehat{\text{CATE}}_{1,3}$ and $\widehat{\text{CATE}}_{2,3}$ based on 5-year survival and 5-year RMST, among 250 simulations under six configurations: (survival times generated from the Weibull distribution with proportional hazards (Weibull-PH) and with nonproportional hazards (Weibull-nPH), and from the log-logistic distribution) $\times$ (10% censoring proportion vs. 40% censoring proportion). Judging on the bases of both patient survival and RMST, our proposed method, riAFT-BART, boasts the smallest bias and variability in the estimation of the treatment effects, regardless of the distribution from which the survival data were generated.

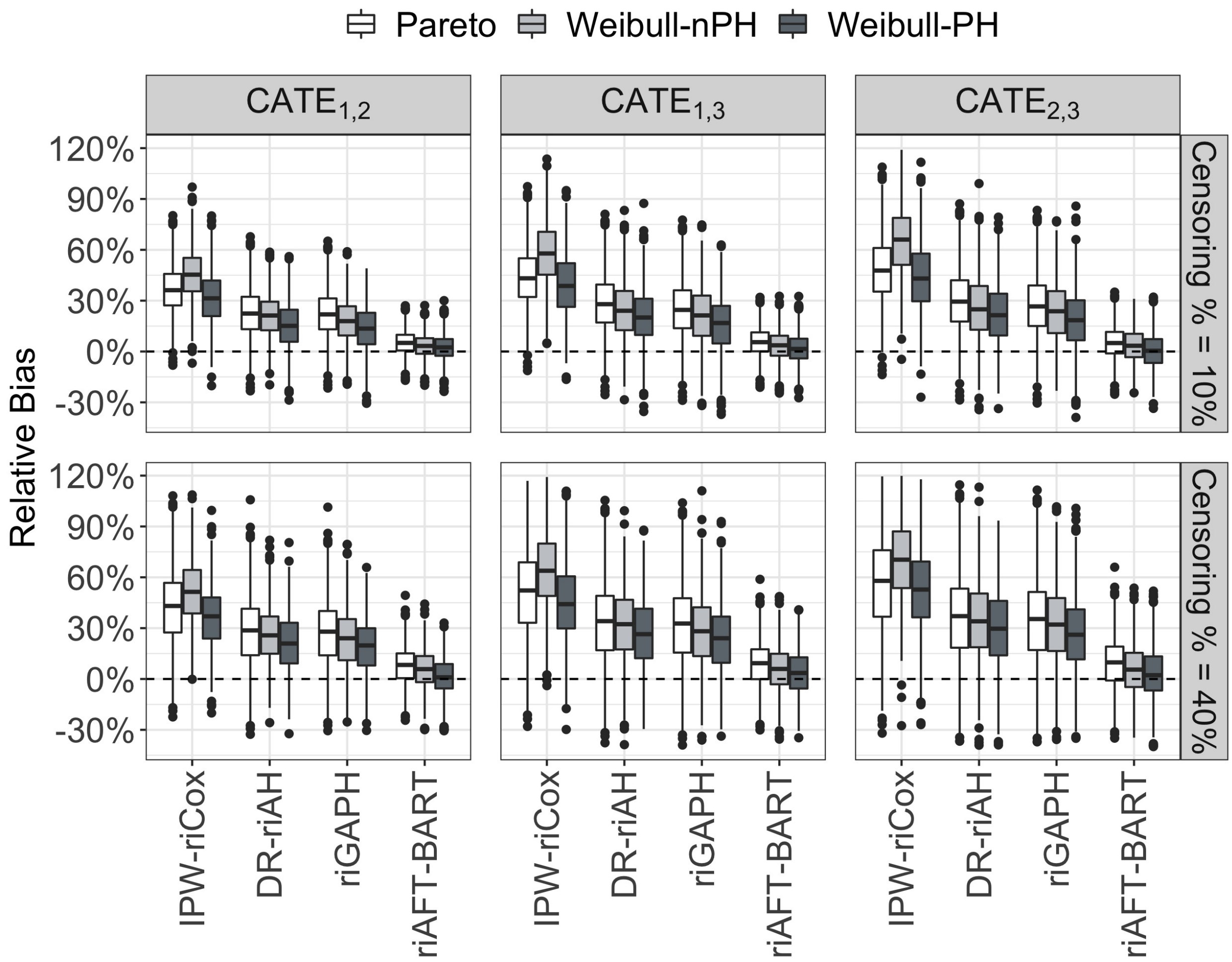

Web Figure 2: Relative biases among 250 replications for each of four methods, IPW-riCox, DR-riAH, riGAPH and riAFT-BART, and three treatment effects $\text{CATE}_{1,2}$, $\text{CATE}_{1,3}$ and $\text{CATE}_{2,3}$ based on 5-year survival under six data configurations: (Weibull-PH, Weibull-nPH, Pareto) $\times$ (10% censoring rate vs. 40% censoring rate). The true treatment effects under Weibull-PH are $\text{CATE}_{1,2}^{0,\text{weibull-PH}} = 0.31$, $\text{CATE}_{1,3}^{0,\text{weibull-PH}} = 0.16$ and $\text{CATE}_{2,3}^{0,\text{weibull-PH}} = -0.15$. The true treatment effects under Weibull-nPH are $\text{CATE}_{1,2}^{0,\text{weibull-nPH}} = 0.32$, $\text{CATE}_{1,3}^{0,\text{weibull-nPH}} = 0.17$ and $\text{CATE}_{2,3}^{0,\text{weibull-nPH}} = -0.15$. The true treatment effects under Pareto are $\text{CATE}_{1,2}^{0,\text{Pareto}} = 0.30$, $\text{CATE}_{1,3}^{0,\text{Pareto}} = 0.15$ and $\text{CATE}_{2,3}^{0,\text{Pareto}} = -0.15$. Censoring %: Censoring proportion.

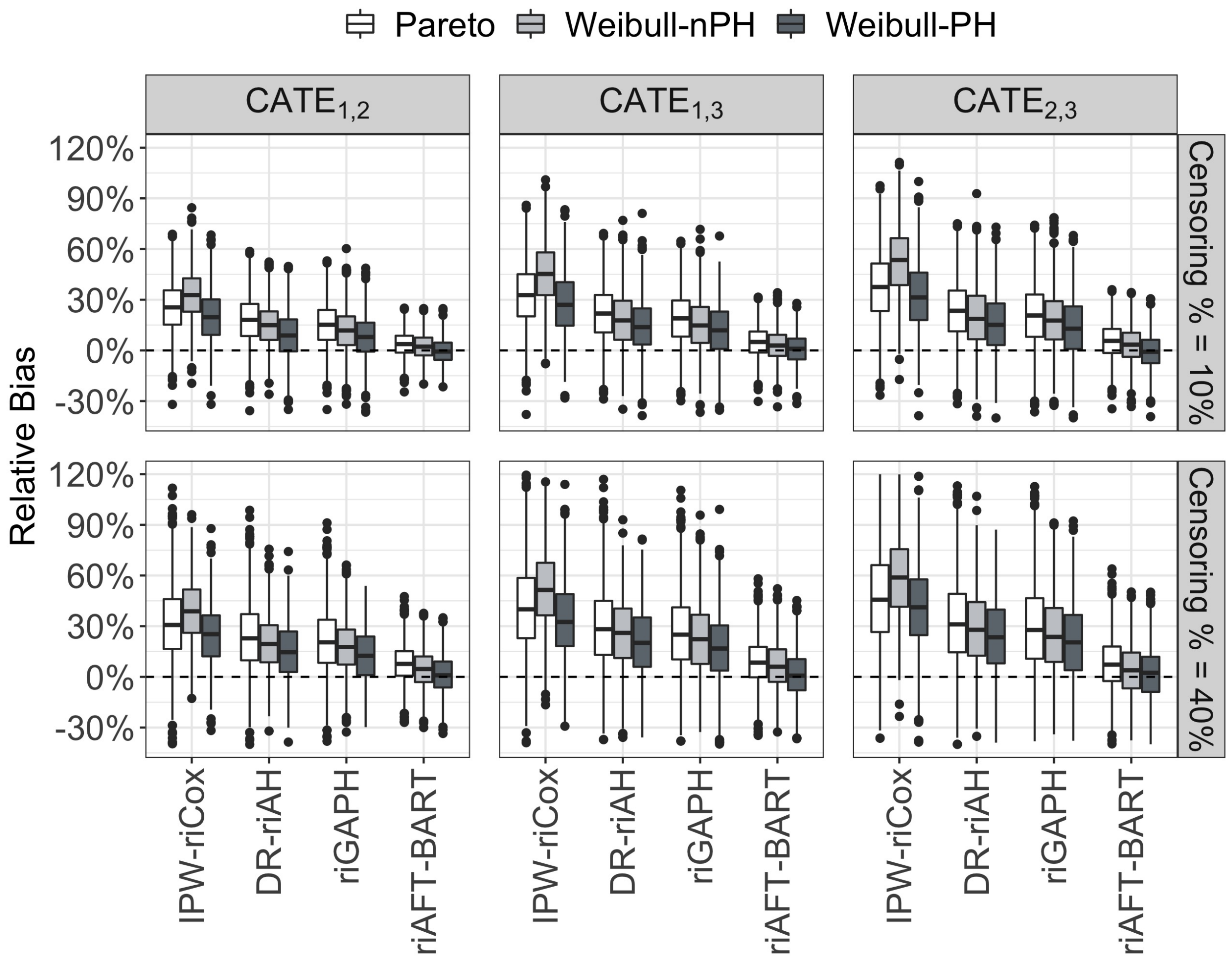

Web Figure 3: Relative biases among 250 replications for each of four methods, IPW-riCox, DR-riAH, riGAPH and riAFT-BART, and three treatment effects $\text{CATE}_{1,2}$, $\text{CATE}_{1,3}$ and $\text{CATE}_{2,3}$ based on 5-year RMST under six data configurations: (Weibull-PH, Weibull-nPH, Pareto) × (10% censoring rate vs. 40% censoring rate). The true treatment effects under Weibull-PH are $\text{CATE}_{1,2}^{0,\text{weibull-PH}} = 7.7$ months, $\text{CATE}_{1,3}^{0,\text{weibull-PH}} = 3.6$ months and $\text{CATE}_{2,3}^{0,\text{weibull-PH}} = -4.1$ months. The true treatment effects under Weibull-nPH are $\text{CATE}_{1,2}^{0,\text{weibull-nPH}} = 8.1$ months, $\text{CATE}_{1,3}^{0,\text{weibull-nPH}} = 3.9$ months and $\text{CATE}_{2,3}^{0,\text{weibull-nPH}} = -4.2$ months. The true treatment effects under Pareto are $\text{CATE}_{1,2}^{0,\text{Pareto}} = 7.6$ months, $\text{CATE}_{1,3}^{0,\text{Pareto}} = 3.7$ months and $\text{CATE}_{2,3}^{0,\text{Pareto}} = -3.9$ months. Censoring %: Censoring proportion.

S3.2 The lognormal survival curves

We generated data adhering to the lognormal AFT model,

$$\log T_{ik}\left(a_j\right) = \boldsymbol{X}_{ik}\beta_{a_j}^{L} + \boldsymbol{G}_{ik}\beta_{a_j}^{NL} + b_k + \epsilon_{ik} \tag{6}$$

for $a_j \in \{1,2,3\}$, where $b_k \sim N(0,4^2), \epsilon_{ik} \sim N(0,1)$ for $a_j = 1,2,3$. Observed and uncensored survival times are generated as $T_{ik} = \sum_{a_j \in \{1,2,3\}} T_{ik}(a_j) I(A_{ik} = a_j)$. We further generate censoring

time C independently from an exponential distribution with the rate parameter selected to induce two different censoring rates: 10% and 40%. Detailed model specifications for outcomes, as in model (6), are given in Table 2. The parameter setup is guided by the NCDB data in our case study so that the resulting Kaplan-Meier survival curves (Web Figure 4) are representative of those in the NCDB data.

Web Figure 5 and Web Figure 6 respectively display boxplots of relative biases in three treatment effect estimates $\widehat{\text{CATE}}_{1,2}$, $\widehat{\text{CATE}}_{1,3}$ and $\widehat{\text{CATE}}_{2,3}$ based on 5-year survival and 5-year RMST, among 250 simulations under six configurations: (Weibull-PH, Weibull-nPH, log normal) $\times$ (10% censoring rate vs. 40% censoring rate). When the survival data adhere to the random-intercept log normal model (6), our proposed method, riAFT-BART achieved the best performance in terms of the bias and precision in estimating the treatment effects. Applying the proposed method to data generated from the Weibull distribution still produced the smallest biases and variability in the treatment effect estimates, among all comparison methods. The difference in the performance of riAFT-BART between the log-normal distribution (normal errors) and the Weibull distribution (extreme-value residuals) is small.

Web Table 2: Coefficients $\beta^L_{a_j}$ and $\beta^{NL}_{a_j}$ of the T-model(6), $a_j \in \{1, 2, 3\}$ to generate potential survival times from the log normal survival curves.

	T-model		
Variables	$a_j = 1$	$a_j = 2$	$a_j = 3$
x_1	0.3	0.3	0.2
x_2	-0.4	0.2	0.3
x_3	-0.3	-0.2	0.2
x_4	0	0	0
x_5	0	0	0
x_6	0.2	-0.4	0.2
x_7	0.3	-0.3	0.5
x_8	0.4	0.3	0.3
x_9	0.5	0.2	0.2
x_{10}	0.6	0.6	0.1
x_1^2	-0.1	-0.4	-0.9
x_1^3	0.1	0.1	0.1
x_2^2	-0.1	-0.3	-0.9
$x_2^2x_5$	0	0	0
x_2x_4	0	0	0
$x_2^2x_4$	0	0	0
x_2x_3	0.3	-0.2	0.3
x_2x_5	0	0	0
$x_2x_3^2$	0.4	-0.2	0.3
$x_2x_4x_5$	0	0	0
$x_1x_2x_3$	-0.1	-0.2	0.2
$\sin(2\pi x_1x_3)$	0	0	0
$\sin(\pi x_1x_3)$	0	0	0
$\sin(\pi x_4x_5)$	0	-0.6	0.5
$\sin(2\pi x_4x_5)$	0.6	0	0

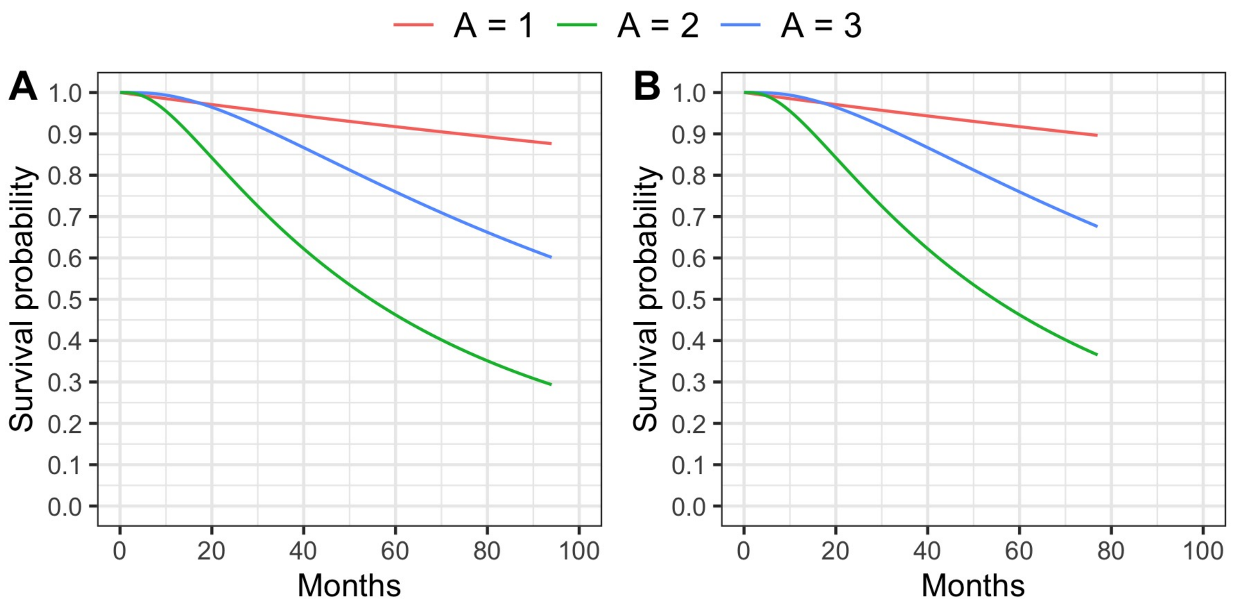

Web Figure 4: Kaplan-Meier survival curves by treatment group. The survival data were generated from the log normal distribution (equation (6)). Panels A represents the scenario of 10% censoring. Panels B represents the scenario of 40% censoring.

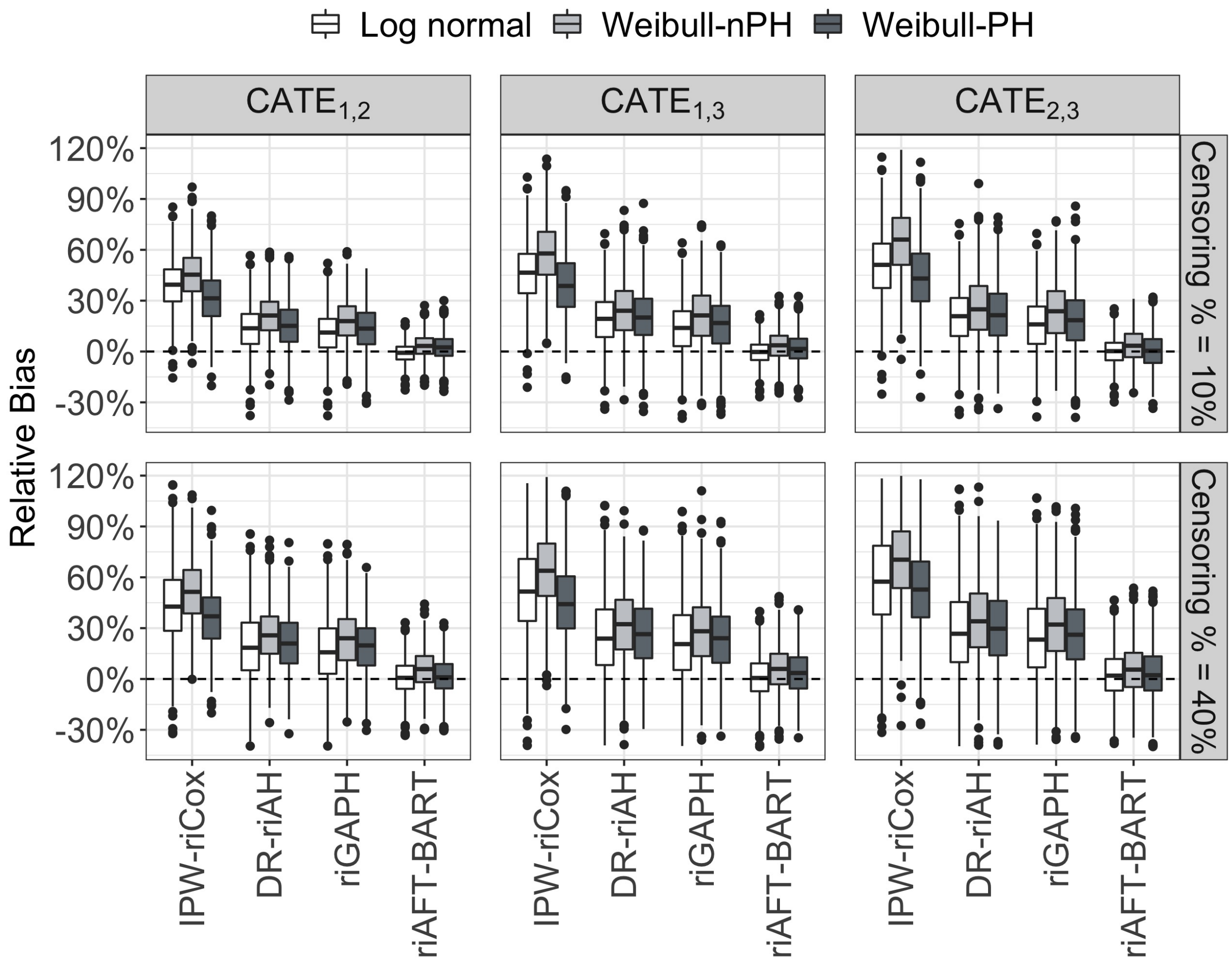

Web Figure 5: Relative biases among 250 replications for each of four methods, IPW-riCox, DR-riAH, riGAPH and riAFT-BART, and three treatment effects $CATE_{1,2}$, $CATE_{1,3}$ and $CATE_{2,3}$ based on 5-year survival under six data configurations: (Weibull-PH, Weibull-nPH, log normal) $\times$ (10% censoring rate vs. 40% censoring rate). The true treatment effects under Weibull-PH are $CATE_{1,2}^{0,\text{weibull-PH}} = 0.31$, $CATE_{1,3}^{0,\text{weibull-PH}} = 0.16$ and $CATE_{2,3}^{0,\text{weibull-PH}} = -0.15$. The true treatment effects under Weibull-nPH are $CATE_{1,2}^{0,\text{weibull-nPH}} = 0.32$, $CATE_{1,3}^{0,\text{weibull-nPH}} = 0.17$ and $CATE_{2,3}^{0,\text{weibull-nPH}} = -0.15$.The true treatment effects under log normal are $CATE_{1,2}^{0,\text{log normal}} = 0.33$, $CATE_{1,3}^{0,\text{log normal}} = 0.17$ and $CATE_{2,3}^{0,\text{log normal}} = -0.16$. Censoring %: Censoring proportion.

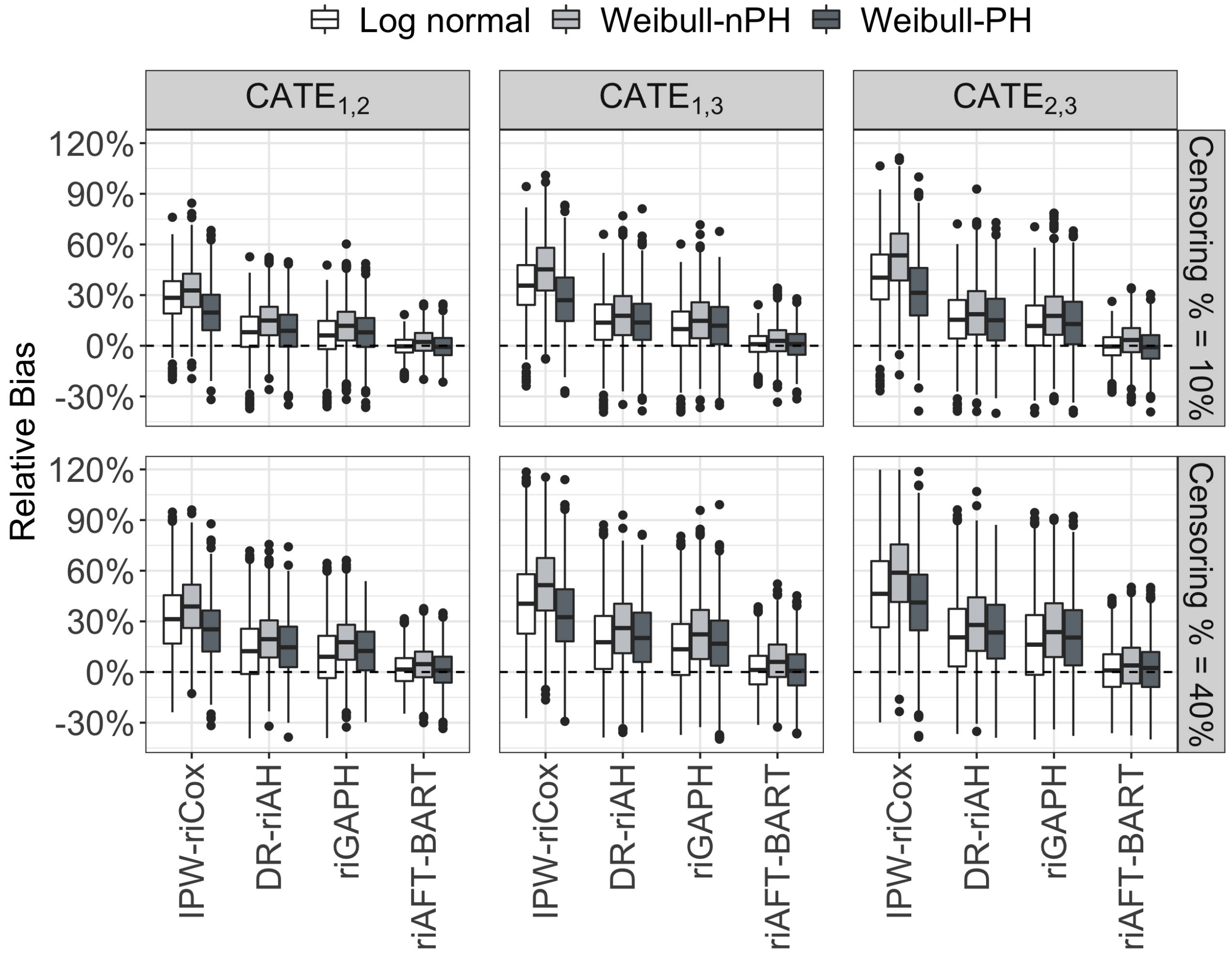

Web Figure 6: Relative biases among 250 replications for each of four methods, IPW-riCox, DR-riAH, riGAPH and riAFT-BART, and three treatment effects $\text{CATE}_{1,2}$, $\text{CATE}_{1,3}$ and $\text{CATE}_{2,3}$ based on 5-year RMST under six data configurations: (Weibull-PH, Weibull-nPH, log normal) $\times$ (10% censoring rate vs. 40% censoring rate). The true treatment effects under Weibull-PH are $\text{CATE}_{1,2}^{0,\text{weibull-PH}} = 7.7$ months, $\text{CATE}_{1,3}^{0,\text{weibull-PH}} = 3.6$ months and $\text{CATE}_{2,3}^{0,\text{weibull-PH}} = -4.1$ months. The true treatment effects under Weibull-nPH are $\text{CATE}_{1,2}^{0,\text{weibull-nPH}} = 8.1$ months, $\text{CATE}_{1,3}^{0,\text{weibull-nPH}} = 3.9$ months and $\text{CATE}_{2,3}^{0,\text{weibull-nPH}} = -4.2$ months.The true treatment effects under log normal are $\text{CATE}_{1,2}^{0,\text{log normal}} = 8.3$ months, $\text{CATE}_{1,3}^{0,\text{log normal}} = 4.0$ months and $\text{CATE}_{2,3}^{0,\text{log normal}} = -4.3$ months. Censoring %: Censoring proportion.

S4 Illustrative simulation for sensitivity analysis approach

We considered a total sample size $N = 10000$ ($n_k = 500, K = 20$) with an unbalanced treatment allocation (the ratio of units = 6:3:1) across three treatment groups, an individual-level binary measured confounder $X_{ik1} \sim \text{Bernoulli}(0.4)$, and an individual-level binary unmeasured confounder $X_{ik2} \sim \text{Bernoulli}(0.5)$. Both treatment assignment and outcome generating mechanisms depend on X_{ik1} and X_{ik2}, but only X_{ik1} is observed. The treatment assignment mechanism follows the

following random-intercept multinomial logistic regression model,

$$\begin{aligned} \ln \frac{P(A_{ik}=1)}{P(A_{ik}=3)} &= 1.6 + 0.2X_{ik1} + 0.4X_{ik2} + \tau_k \\ \ln \frac{P(A_{ik}=2)}{P(A_{ik}=3)} &= -0.2 + 0.3X_{ik1} - 0.3X_{ik2} + \tau_k, \end{aligned} \tag{7}$$

where $\tau_k \sim N(0, 1^2)$. Three sets of non-parallel response surfaces are generated as

$$T_{ik}(a_j) = \begin{cases} \left[\dfrac{-\log U}{6\exp\left(-0.8X_{ik1} - 1.2X_{ik2} + b_k\right)}\right]^{1/2}, & \text{if } a_j = 1 \\ \left[\dfrac{-\log U}{2\exp\left(-0.5X_{ik1} - 2.2X_{ik2} + b_k\right)}\right]^{1/2}, & \text{if } a_j = 2 \\ \left[\dfrac{-\log U}{4\exp\left(-0.3X_{ik1} + 1.0X_{ik2} + b_k\right)}\right]^{1/2}, & \text{if } a_j = 3 \end{cases} \tag{8}$$

where U is a random variable following the uniform distribution on the interval $[0, 1]$ and $b_k \sim N(0, 4^2)$. Observed and uncensored survival times are generated as $T_{ik} = \sum_{a_j \in \{1,2,3\}} T_{ik}(a_j) I(A_{ik} = a_j)$. Finally, we generate the censoring time C independently from an exponential distribution with the rate parameter selected to induce 10% censoring.

Under this simulation configuration, the true $\text{CATE}^0_{1,2} = 0.31$, $\text{CATE}^0_{1,3} = 0.21$ and $\text{CATE}^0_{2,3} = -0.10$ in terms of the log survival time in months.

S5 Supplementary Tables and Figures

Web Table 3: Interpretation of assumed priors on $c(a_j, a_{j'}, \mid \boldsymbol{x}, v)$ and $c(a_{j'}, a_j \mid \boldsymbol{x}, v)$ for average treatment effect based on log survival time.

Prior assumption		Interpretation and implications of the assumptions
$c(a_j, a_{j'} \mid \boldsymbol{x}, v)$	$c(a_{j'}, a_j \mid \boldsymbol{x}, v)$	
> 0	< 0	Individuals treated with a_j will on average have longer potential survival time to both a_j and $a_{j'}$ than individuals treated with $a_{j'}$; i.e. healthier individuals are treated with a_j.
< 0	> 0	Contrary to the above interpretation, unhealthier individuals are treated with a_j.
< 0	< 0	The potential survival time to $a_j(a_{j'})$ is shorter among those who choose it than among those who choose $a_{j'}(a_j)$. Thus, the observed treatment allocation between these two approaches is undesirable relative to the alternative which reverses treatment assignment for everyone.
> 0	> 0	Contrary to the above interpretation, the observed treatment allocation between these two approaches is beneficial relative to the alternative which reverses treatment assignment for everyone.

Web Table 4: Specifications of treatment assignment model (A-model) in equation (8) and outcome generating model (T-model) in equation (10); and coefficients ξ_1^L, ξ_2^L and ξ_1^{NL}, ξ_2^{NL} of the A-model and $\beta_{a_j}^L$ and $\beta_{a_j}^{NL}$ of the T-model, $a_j \in \{1,2,3\}$. We set $\xi_{01} = 0.9$ and $\xi_{02} = -1.0$ to generate the 6:3:1 ratio of unit across three treatment groups.

	A-model		T-model		
Variables	$a_j = 1$	$a_j = 2$	$a_j = 1$	$a_j = 2$	$a_j = 3$
x_1	0	0	1.2	1.0	0.9
x_2	0.3	0.3	1.0	0.8	0.9
x_3	0	0	1.2	1.0	0.9
x_4	0.5	1.2	0	0	0
x_5	0.4	1.1	0	0	0
x_6	0.2	0.9	1.2	1.0	0.4
x_7	0.3	0.5	0.6	0.8	0.5
x_8	1.1	0.6	0.4	0.4	0.3
x_9	0.6	0.7	0.5	0.6	0.2
x_{10}	1.2	0.6	0.6	0.6	0.1
x_1^2	0	0	0.9	0.7	0.85
x_1^3	0	0	0.4	0.5	0.3
x_2^2	0.4	0.75	0.3	0.8	0.7
$x_2^2 x_5$	0.4	0.8	0	0	0
$x_2 x_4$	0.5	0.9	0	0	0
$x_2^2 x_4$	0.8	0.7	0	0	0
$x_2 x_3$	0	0	0.3	0.4	0.8
$x_2 x_5$	0.7	0.7	0	0	0
$x_2 x_3^2$	0	0	0.4	0.7	0.8
$x_2 x_4 x_5$	0.5	0.4	0	0	0
$x_1 x_2 x_3$	0	0	0.3	0.5	0.7
$\sin(2\pi x_1 x_3)$	-0.8	0	0	0	0
$\sin(\pi x_1 x_3)$	0	-0.5	0	0	0
$\sin(\pi x_4 x_5)$	0	0	0	0.6	0.5
$\sin(2\pi x_4 x_5)$	0	0	0.6	0	0

Web Table 5: The coverage probability for three treatment effect estimates $\widehat{CATE}_{1,2}$, $\widehat{CATE}_{1,3}$ and $\widehat{CATE}_{2,3}$ based on 5-year survival probability under four data configurations: (proportional hazards vs. nonproporitonal hazards) × (10% censoring proportion vs. 40% censoring proportion).

		Proportional hazards			Nonproportional hazards		
Censoring %	Methods	$\text{CATE}_{1,2}$	$\text{CATE}_{1,3}$	$\text{CATE}_{2,3}$	$\text{CATE}_{1,2}$	$\text{CATE}_{1,3}$	$\text{CATE}_{2,3}$
10%	IPW-riCox	16.0	20.4	18.4	7.6	12.4	10.4
	DR-riAH	77.2	78.0	77.6	76.0	76.4	75.2
	ri-GAPH	79.4	80.0	80.4	77.2	79.2	78.0
	riAFT-BART	95.6	94.8	94.8	93.6	93.6	94.0
40%	IPW-riCox	14.0	18.8	16.8	6.0	10.8	8.4
	DR-riAH	73.2	75.6	74.4	73.6	73.2	72.0
	ri-GAPH	76.8	78.8	77.6	74.4	77.6	75.2
	riAFT-BART	94.8	94.4	95.2	92.8	93.2	93.6

Web Table 6: Assessing the performance of the estimation of f in model (1), using the mean bias (MB) and root-mean-squared error (RMSE) in the posterior mean of f across 250 data replications under four data configurations: (proportional hazards vs. nonproporitonal hazards) × (10% censoring rate vs. 40% censoring rate), for three treatment groups: $A = 1, 2, 3$. The true values of the f function under proportional hazards are $f^{0,\text{PH}}_{A=1} = -6.242$, $f^{0,\text{PH}}_{A=2} = -5.691$ and $f^{0,\text{PH}}_{A=3} = -5.909$. The values of the f function under nonproportional hazards are $f^{0,\text{nPH}}_{A=1} = -6.489$, $f^{0,\text{nPH}}_{A=2} = -5.965$ and $f^{0,\text{nPH}}_{A=3} = -6.325$. PH= proportional hazards; nPH=non proportional hazards. Censoring %: Censoring proportion.

		PH		nPH	
Censoring %	Tretament	MB	RMSE	MB	RMSE
10%	A = 1	-0.027	0.195	-0.116	0.242
10%	A = 2	-0.024	0.187	-0.089	0.229
10%	A = 3	-0.045	0.227	-0.132	0.262
40%	A = 1	-0.134	0.317	-0.224	0.367
40%	A = 2	-0.104	0.276	-0.153	0.325
40%	A = 3	-0.087	0.297	-0.200	0.390

Web Table 7: The mean bias (MB) and root-mean-squared-error (RMSE) in the average treatment effects estimated from the random-effects model riAFT-BART and the fixed-effects model AFT-BART, based on 5-year RMST across 250 data replications under four data configurations: (proportional hazards vs. nonproporitonal hazards) × (10% censoring rate vs. 40% censoring rate). The number of trees was set to 200 for riAFT-BART and 300 for AFT-BART to pick up an additional covariate. The survival times were generated from the Weibull distribution. The true treatment effects under proportional hazards are $\text{CATE}_{1,2}^{0,\text{PH}} = 7.7$ months, $\text{CATE}_{1,3}^{0,\text{PH}} = 3.6$ months and $\text{CATE}_{2,3}^{0,\text{PH}} = -4.1$ months. The true treatment effects under nonproportional hazards are $\text{CATE}_{1,2}^{0,\text{nPH}} = 8.1$ months, $\text{CATE}_{1,3}^{0,\text{nPH}} = 3.9$ months and $\text{CATE}_{2,3}^{0,\text{nPH}} = -4.2$ months. Censoring %: Censoring proportion.

			Proportional hazards		Nonproporitonal hazards	
Censoring %	Causal estimand	Method	MB	RMSE	MB	RMSE
10%	$\text{CATE}_{1,2}$	riAFT-BART	-0.038	0.597	0.180	0.637
10%	$\text{CATE}_{1,2}$	AFT-BART	-0.032	0.590	0.178	0.632
10%	$\text{CATE}_{1,3}$	riAFT-BART	0.029	0.353	0.117	0.366
10%	$\text{CATE}_{1,3}$	AFT-BART	0.028	0.349	0.115	0.363
10%	$\text{CATE}_{2,3}$	riAFT-BART	0.027	0.446	-0.139	0.457
10%	$\text{CATE}_{2,3}$	AFT-BART	0.022	0.440	-0.136	0.454
40%	$\text{CATE}_{1,2}$	riAFT-BART	0.091	0.917	0.360	0.965
40%	$\text{CATE}_{1,2}$	AFT-BART	0.085	0.899	0.355	0.932
40%	$\text{CATE}_{1,3}$	riAFT-BART	0.039	0.529	0.244	0.592
40%	$\text{CATE}_{1,3}$	AFT-BART	0.036	0.519	0.240	0.571
40%	$\text{CATE}_{2,3}$	riAFT-BART	-0.078	0.647	-0.146	0.668
40%	$\text{CATE}_{2,3}$	AFT-BART	-0.074	0.637	-0.142	0.664

Web Table 8: The mean bias (MB) and root-mean-squared-error (RMSE) in the average treatment effects estimated from the random-effects model riAFT-BART and the fixed-effects model AFT-BART, based on 5-year survival across 250 data replications under four data configurations: (proportional hazards vs. nonproporitonal hazards) × (10% censoring rate vs. 40% censoring rate). The number of trees was set to 200 for riAFT-BART and 300 for AFT-BART to pick up an additional covariate. The survival times were generated from the Weibull distribution. The survival times were generated from the Weibull distribution. The true treatment effects under proportional hazards are $\text{CATE}_{1,2}^{0,\text{PH}} = 0.31$, $\text{CATE}_{1,3}^{0,\text{PH}} = 0.16$ and $\text{CATE}_{2,3}^{0,\text{PH}} = -0.15$. The true treatment effects under nonproportional hazards are $\text{CATE}_{1,2}^{0,\text{nPH}} = 0.32$, $\text{CATE}_{1,3}^{0,\text{nPH}} = 0.17$ and $\text{CATE}_{2,3}^{0,\text{nPH}} = -0.15$. Censoring %: Censoring proportion.

			Proportional hazards		Nonproporitonal hazards	
Censoring %	Causal estimand	Method	MB	RMSE	MB	RMSE
10%	$\text{CATE}_{1,2}$	riAFT-BART	0.007	0.024	0.010	0.024
10%	$\text{CATE}_{1,2}$	AFT-BART	0.007	0.024	0.010	0.024
10%	$\text{CATE}_{1,3}$	riAFT-BART	0.003	0.014	0.005	0.015
10%	$\text{CATE}_{1,3}$	AFT-BART	0.003	0.014	0.005	0.014
10%	$\text{CATE}_{2,3}$	riAFT-BART	-0.001	0.015	-0.006	0.016
10%	$\text{CATE}_{2,3}$	AFT-BART	-0.001	0.015	-0.006	0.016
40%	$\text{CATE}_{1,2}$	riAFT-BART	0.004	0.034	0.017	0.038
40%	$\text{CATE}_{1,2}$	AFT-BART	0.002	0.033	0.015	0.036
40%	$\text{CATE}_{1,3}$	riAFT-BART	0.005	0.021	0.009	0.022
40%	$\text{CATE}_{1,3}$	AFT-BART	0.004	0.020	0.008	0.021
40%	$\text{CATE}_{2,3}$	riAFT-BART	-0.004	0.024	-0.008	0.024
40%	$\text{CATE}_{2,3}$	AFT-BART	-0.004	0.023	-0.008	0.023

Web Table 9: Specifications of the "linear" outcome generating model (T-model) in equation (10) of Section 4.2. Only the untransformed versions of the confounders $\boldsymbol{X}_{ik}$ were included. The values of the parameters were chosen to generate the similar Kaplan-Meier survival curves as in the NCDB dataset.

	Weibull T-model		
Variables	$a_j = 1$	$a_j = 2$	$a_j = 3$
x_1	1.2	1.0	0.9
x_2	1.0	0.8	0.9
x_3	1.0	1.0	0.9
x_4	0.4	0.2	0.3
x_5	0.8	0.1	0.3
x_6	1.2	1.0	0.4
x_7	0.6	0.8	0.5
x_8	0.9	1.4	1.3
x_9	1.5	1.6	1.2
x_{10}	1.6	1.6	1.1

Web Table 10: Descriptions of pre-treatment variables and hospital locations (clusters) for each of three treatment groups in NCDB data.

Characteristics	Overall $N = 23058$	RP $N = 14237$	EBRT+AD $N = 6683$	EBRT+brachy±AD $N = 2138$
Age (years), mean (SD)	71.14 (4.35)	69.78 (3.23)	73.69 (5.09)	72.20 (4.42)
Race, N (%)				
White	19583 (84.9)	12335 (86.6)	5527 (82.7)	1721 (80.5)
Black	3840 (14.1)	1311 (9.2)	922 (13.8)	317 (14.8)
American Indian, Aleutian, or Eskimo	925 (4.0)	591 (4.2)	234 (3.5)	100 (4.7)
Spanish or Hispanic Origin, N (%)				
Non-Spanish; non-Hispanic	22086 (95.8)	13649 (95.9)	6385 (95.5)	2052 (96.0)
Spanish or Hispanic	972 (4.2)	588 (4.1)	298 (4.1)	86 (4.0)
Insurance, N (%)				
Yes	22891 (99.3)	14132 (99.3)	6629 (99.2)	2130 (98.8)
No	167 (0.7)	105 (0.7)	54 (0.8)	8 (0.4)
Income, N (%)				
<$30,000	2464 (10.7)	1462 (10.3)	755 (11.3)	247 (11.6)
$30,000 - $34,999	3936 (17.1)	2408 (16.9)	1171 (17.5)	357 (16.7)
$35,000 - $45,999	6445 (28.0)	3977 (27.9)	1927 (28.8)	541 (25.3)
>$46,000	10213 (44.3)	6390 (44.9)	2830 (42.3)	993 (46.4)
Education, N (%)				
<14%	9501 (41.2)	6161 (43.3)	2464 (36.9)	876 (41.0)
14%-19.9%	5663 (24.6)	3397 (23.9)	1753 (26.2)	513 (24.0)
20%-28.9%	4838 (21.0)	2852 (20.0)	1546 (23.1)	440 (20.6)
>28.9%	3056 (13.3)	1827 (12.8)	920 (13.8)	309 (14.5)
Clinical T Stage, N (%)				
≤cT2	20509 (88.9)	12972 (91.1)	5699 (85.3)	1838 (86.0)
≥cT3	2549 (11.1)	1265 (8.9)	984 (14.7)	300 (14.0)
Year of Diagnosis, N (%)				
2004-2010	2889 (12.5)	1825 (12.8)	670 (10.0)	394 (18.4)
2011	3353 (14.5)	2069 (14.5)	859 (12.9)	425 (19.9)
2012	3250 (14.1)	2048 (14.4)	892 (13.3)	310 (14.5)
2013	3949 (17.1)	2501 (17.6)	1165 (17.4)	283 (13.2)
2014	4292 (18.6)	2549 (17.9)	1418 (21.2)	325 (15.2)
2015	5325 (23.1)	3245 (22.8)	1679 (25.1)	401 (18.8)
PSA (ng/mL), mean (SD)	17.11 (19.86)	15.91 (19.01)	19.77 (21.60)	16.80 (18.90)
Gleason score, N (%)				
6	1016 (4.4)	783 (5.5)	139 (2.1)	94 (4.4)
7	3245 (14.1)	1961 (13.8)	937 (14.0)	347 (16.2)
8	11116 (48.2)	7079 (49.7)	3041 (45.5)	996 (46.6)
9	7142 (31.0)	4175 (29.3)	2312 (34.6)	655 (30.6)
10	539 (2.3)	239 (1.7)	254 (3.8)	46 (2.2)
Location, N (%)				
East North Central	1020 (4.4)	755 (5.3)	177 (2.6)	88 (4.1)
East South Central	1850 (8.0)	1367 (9.6)	312 (4.7)	171 (8.0)
Middle Atlantic	3558 (15.4)	1775 (12.5)	1469 (22.0)	314 (14.7)
Mountain	1594 (6.9)	731 (5.1)	822 (12.3)	41 (1.9)
New England	4113 (17.8)	2521 (17.7)	1229 (18.4)	363 (17.0)
Pacific	2910 (12.6)	2049 (14.4)	594 (8.9)	267 (12.5)
South Atlantic	4760 (20.6)	2520 (17.7)	1505 (22.5)	735 (34.4)
West North Central	2037 (8.8)	1566 (11.0)	353 (5.3)	118 (5.5)
West South Central	1216 (5.3)	953 (6.7)	222 (3.3)	41 (1.9)

Abbreviations: SD = standard deviation; PSA: Prostate-Specific Antigen

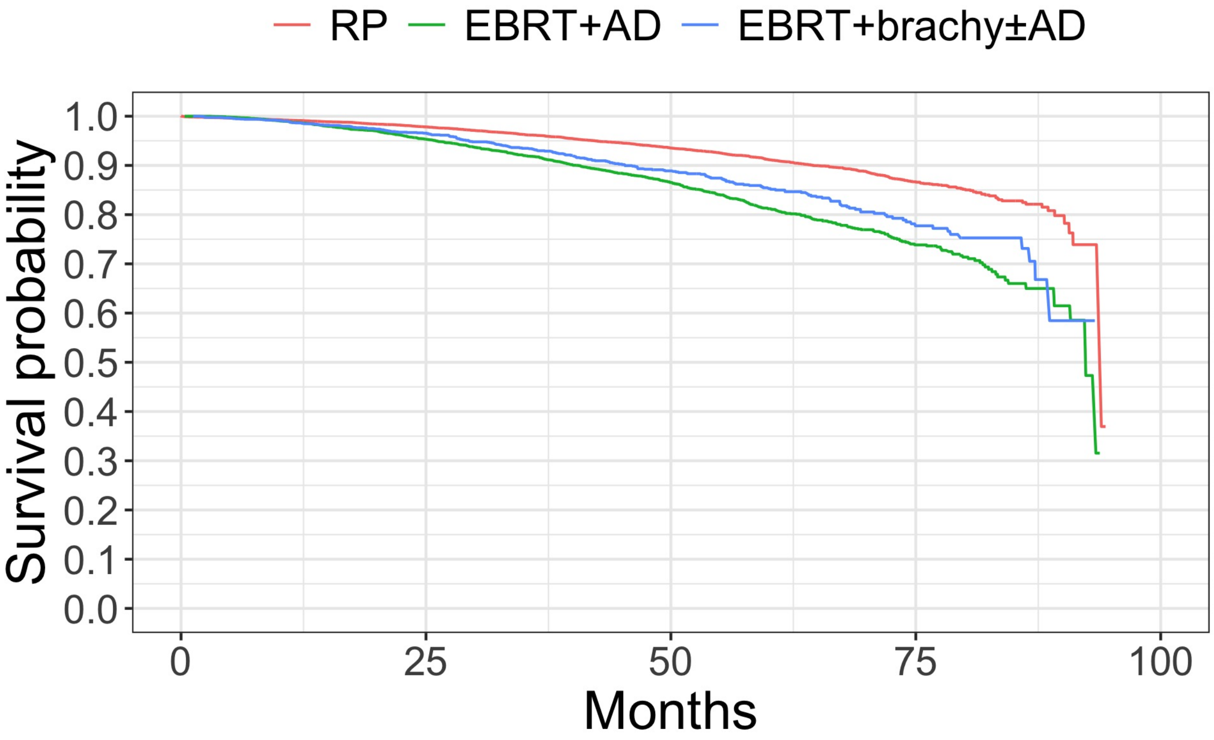

Web Figure 7: The Kaplan-Meier survival curves for three treatment groups: radical prostatectomy (RP), external beam radiotherapy combined with androgen deprivation (EBRT+AD), and external beam radiotherapy plus brachytherapy with or without androgen deprivation (EBRT+brachy±AD), in NCDB data.

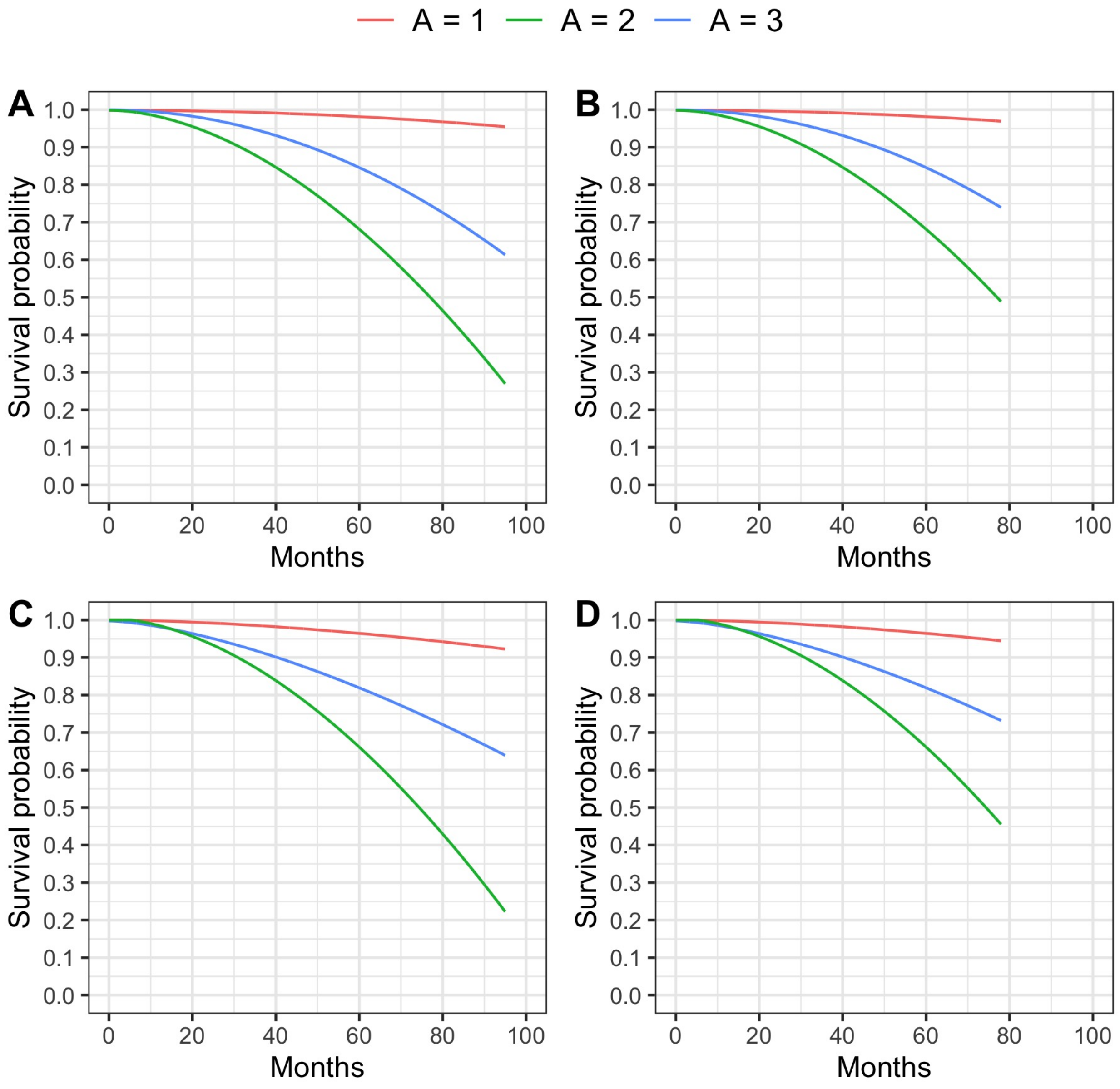

Web Figure 8: Kaplan-Meier survival curves for three treatment groups generated in our simulation study in Section 4. Panels A–D respectively represent scenarios corresponding to proportional hazards with 10% censoring, proportional hazards with 40% censoring, non proportional hazards with 10% censoring and non proportional hazards with 40% censoring.

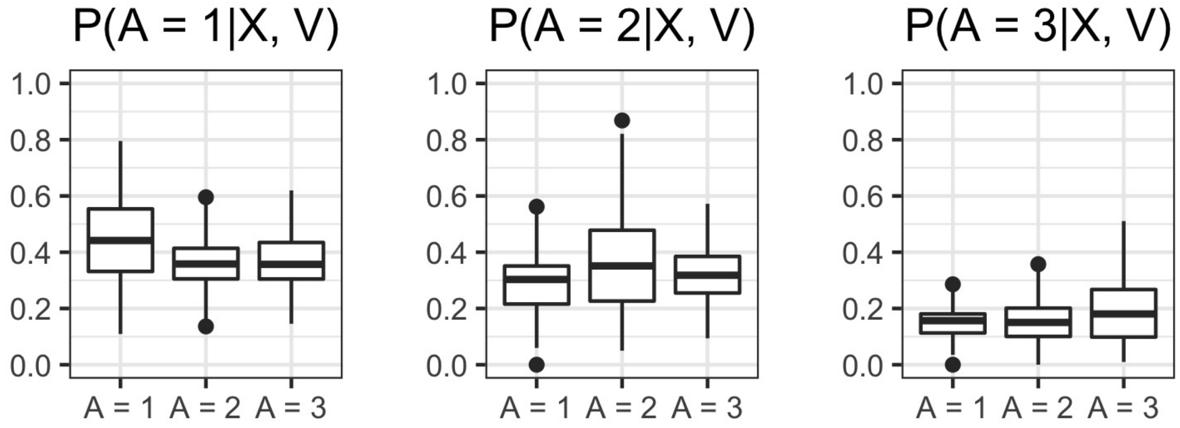

Web Figure 9: Overlap assessment for data simulated under treatment assignment model in equation (8). Each panel presents boxplots by treatment group of the true generalized propensity scores for one of three treatments, and for every unit in the sample. The left panel presents treatment 1, the middle panel presents treatment 2, and the right panel presents treatment 3.

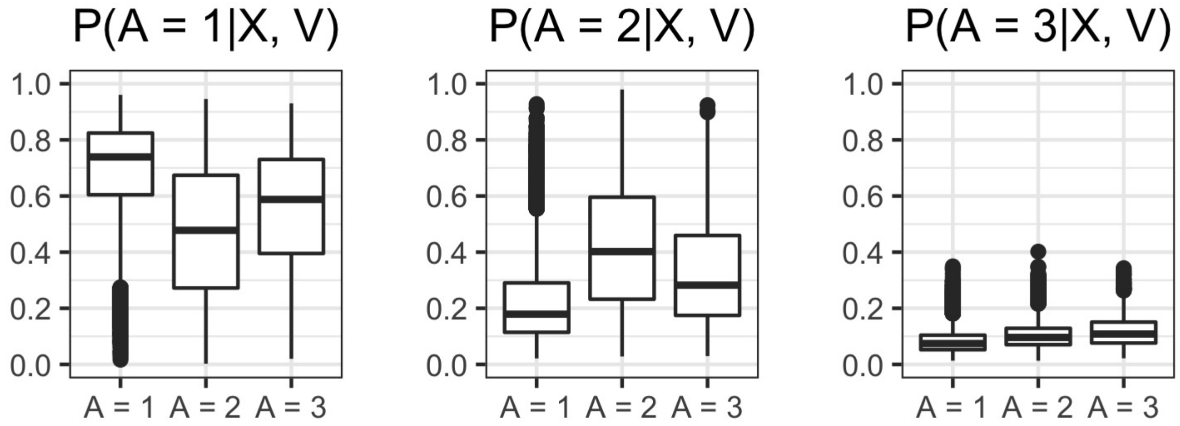

Web Figure 10: Overlap assessment for three treatment groups in the NCDB data. Each panel presents boxplots by treatment group of the generalized propensity scores, estimated by Super Learner, for one of three treatments, and for every individual in the sample. The left panel presents treatment 1 = radical prostatectomy (RP), the middle panel presents treatment 2 = external beam radiotherapy combined with androgen deprivation (EBRT+AD), and the right panel presents treatment 3 = external beam radiotherapy plus brachytherapy with or without androgen deprivation (EBRT+brachy±AD).

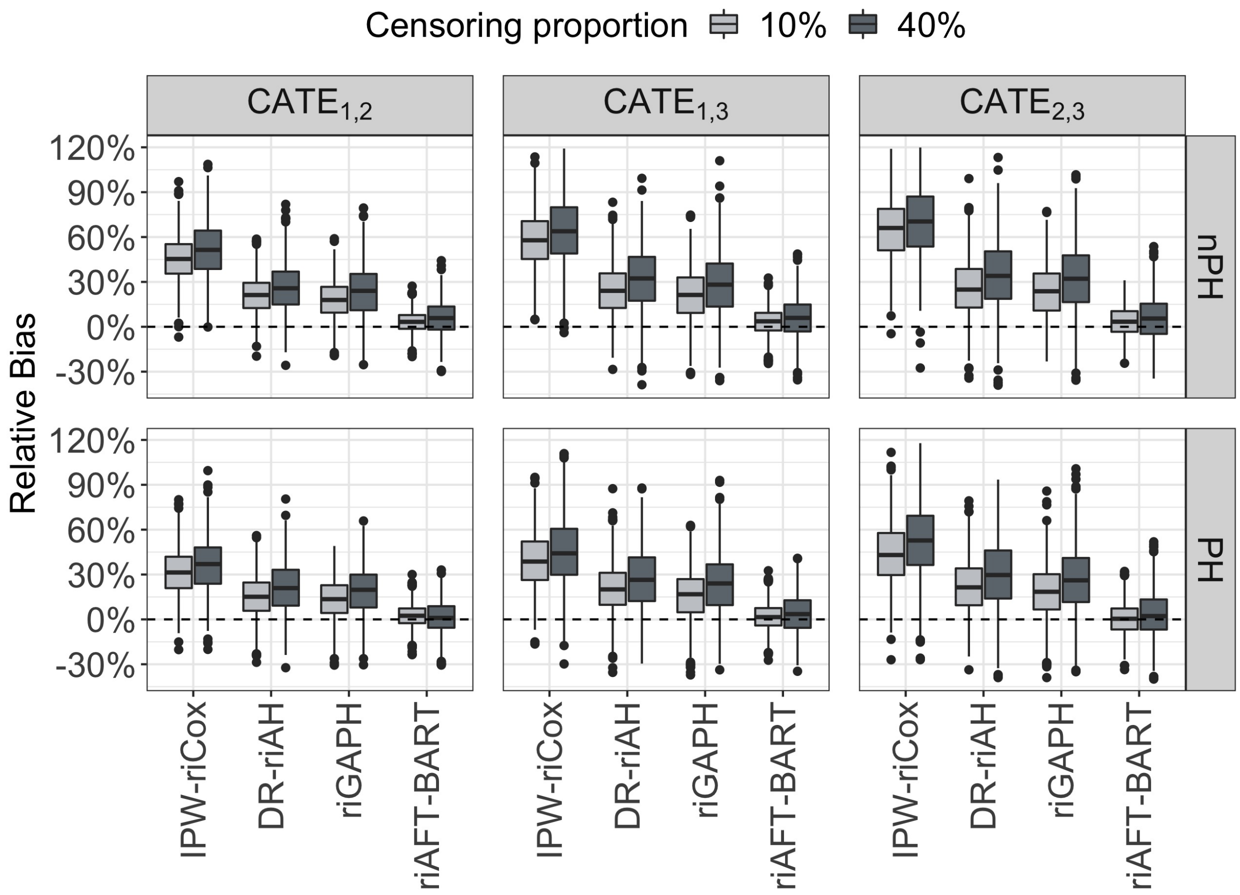

Web Figure 11: Relative biases among 250 replications for each of four methods, IPW-riCox, DR-riAH, riGAPH and riAFT-BART, and three treatment effects $CATE_{1,2}$, $CATE_{1,3}$ and $CATE_{2,3}$ based on 5-year survival under four data configurations: (proportional hazards vs. nonproporitonal hazards) × (10% censoring proportion vs. 40% censoring proportion). The true treatment effects under proportional hazards are $CATE^{0,PH}_{1,2} = 0.31$, $CATE^{0,PH}_{1,3} = 0.16$ and $CATE^{0,PH}_{2,3} = -0.15$. The true treatment effects under nonproportional hazards are $CATE^{0,nPH}_{1,2} = 0.32$, $CATE^{0,nPH}_{1,3} = 0.17$ and $CATE^{0,nPH}_{2,3} = -0.15$.

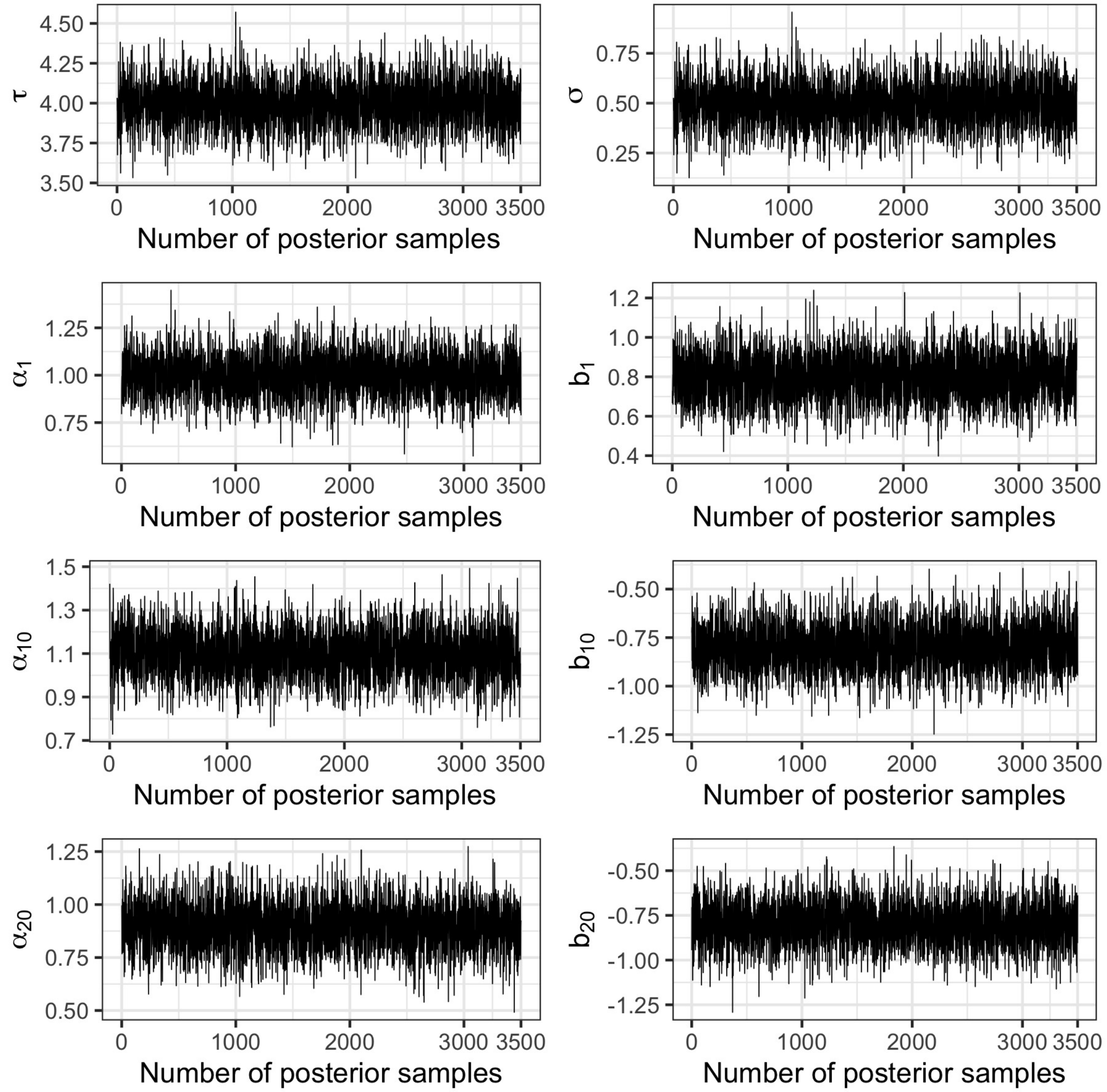

Web Figure 12: Assessing convergence of the chain by plotting 3500 posterior draws of the variance parameters τ and σ, and cluster-specific parameter α_k and the random intercepts b_k for clusters $k = 1$, $k = 10$ and $k = 20$.

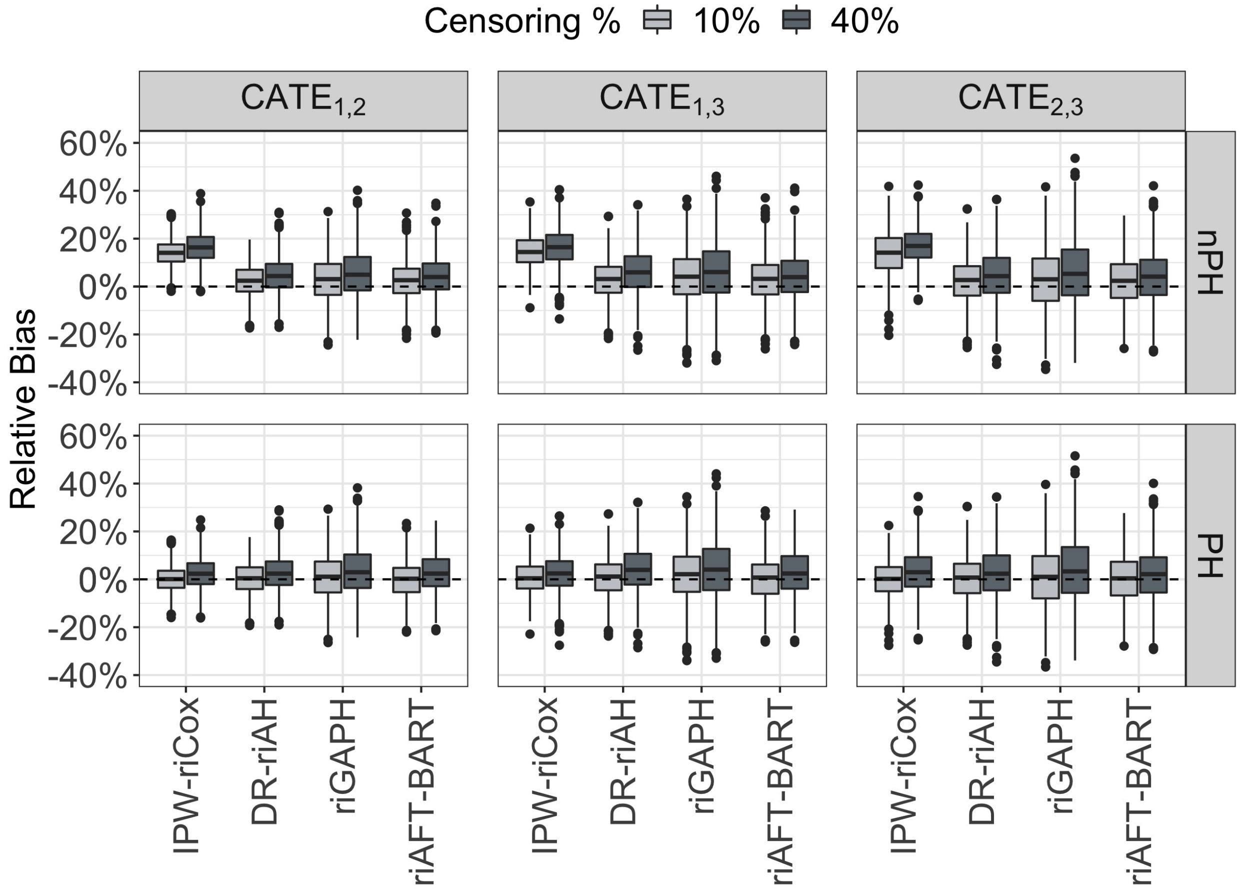

Web Figure 13: Relative biases among 250 replications for each of four methods, IPW-riCox, DR-riAH, riGAPH and riAFT-BART, and three treatment effects $\text{CATE}_{1,2}$, $\text{CATE}_{1,3}$ and $\text{CATE}_{2,3}$ based on 5-year survival under four data configurations: (proportional hazards vs. nonproporitonal hazards) $\times$ (10% censoring rate vs. 40% censoring rate). The data is generated from Weibull distribution without the nonlinear terms in the outcome model. The true treatment effects under proportional hazards are $\text{CATE}^{0,\text{PH}}_{1,2} = 0.31$, $\text{CATE}^{0,\text{PH}}_{1,3} = 0.16$ and $\text{CATE}^{0,\text{PH}}_{2,3} = -0.15$. The true treatment effects under nonproportional hazards are $\text{CATE}^{0,\text{nPH}}_{1,2} = 0.32$, $\text{CATE}^{0,\text{nPH}}_{1,3} = 0.17$ and $\text{CATE}^{0,\text{nPH}}_{2,3} = -0.15$.

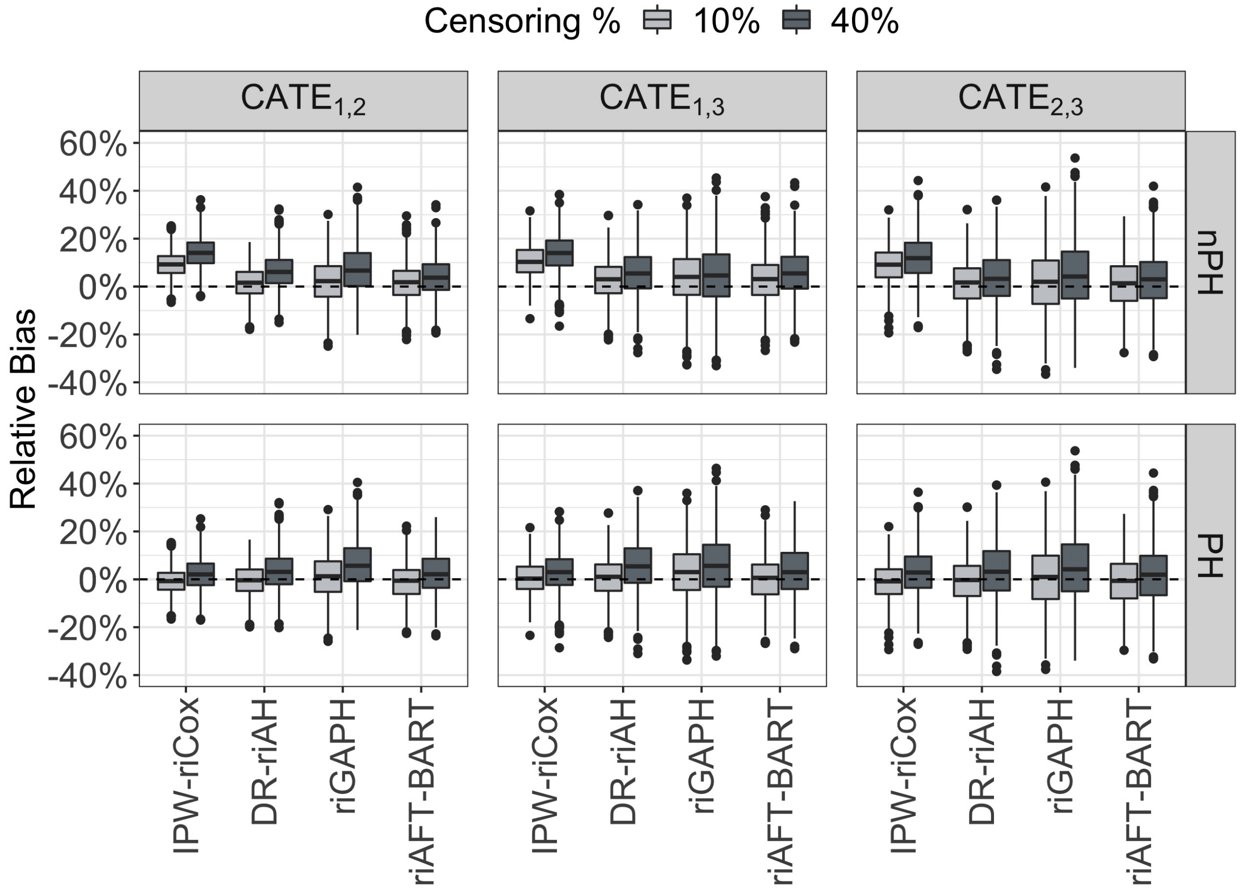

Web Figure 14: Relative biases among 250 replications for each of four methods, IPW-riCox, DR-riAH, riGAPH and riAFT-BART, and three treatment effects $\text{CATE}_{1,2}$, $\text{CATE}_{1,3}$ and $\text{CATE}_{2,3}$ based on 5-year RMST under four data configurations: (proportional hazards vs. nonproporitonal hazards) $\times$ (10% censoring rate vs. 40% censoring rate). The data is generated from Weibull distribution without the nonlinear terms in the outcome model. The true treatment effects under proportional hazards are $\text{CATE}_{1,2}^{0,\text{PH}} = 7.7$ months, $\text{CATE}_{1,3}^{0,\text{PH}} = 3.6$ months and $\text{CATE}_{2,3}^{0,\text{PH}} = -4.1$ months. The true treatment effects under nonproportional hazards are $\text{CATE}_{1,2}^{0,\text{nPH}} = 8.1$ months, $\text{CATE}_{1,3}^{0,\text{nPH}} = 3.9$ months and $\text{CATE}_{2,3}^{0,\text{nPH}} = -4.2$ months.

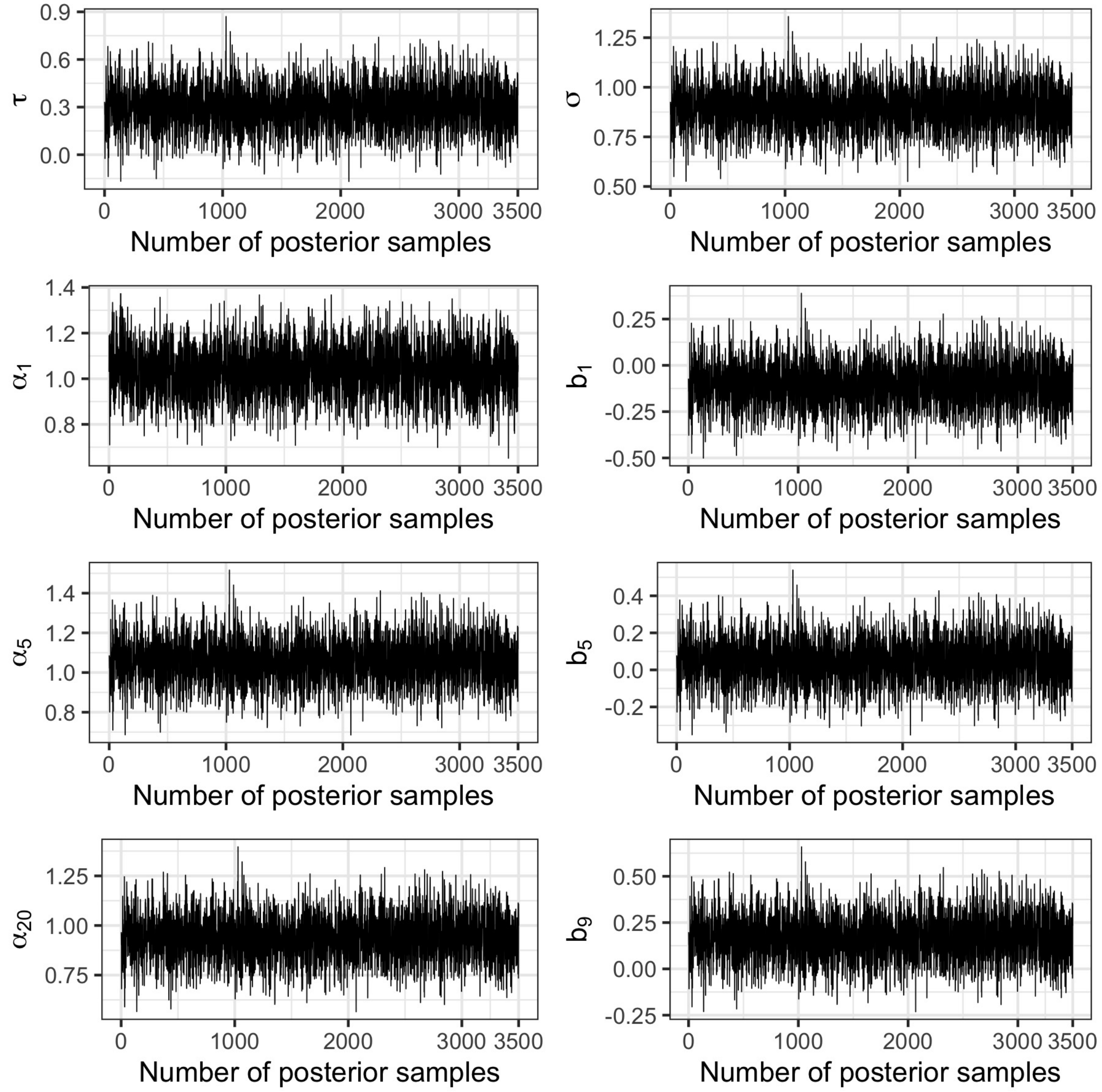

Web Figure 15: Assessing convergence of the chain by plotting 3500 posterior draws of the variance parameters τ and σ, the cluster-specific parameter α_k and the random intercepts b_k for clusters $k = 1$, $k = 5$ and $k = 9$ in NCDB data.

References

Bender, R., Augustin, T., and Blettner, M. (2005). Generating survival times to simulate cox proportional hazards models. *Statistics in Medicine* **24,** 1713–1723.

Hu, L., Zou, J., Gu, C., Ji, J., Lopez, M., and Kale, M. (2022). A flexible sensitivity analysis approach for unmeasured confounding with multiple treatments and a binary outcome with application to seer-medicare lung cancer data. *The Annals of Applied Statistics* In press.
